# Tower-to-global upscaling of terrestrial carbon fluxes driven by MODIS-LAI, Sentinel-3-LAI and ERA5-Land data

**DOI:** 10.1016/j.ecolind.2025.113597

**Published:** 2025-08

**Authors:** Pablo Reyes-Muñoz, Dávid D.Kovács, Jochem Verrelst

**Affiliations:** Image Processing Laboratory (IPL), University of Valencia, C/Catedrático Agustín Escardino Benlloch 9, Paterna, 46980 Valencia, Spain

**Keywords:** Carbon cycle, Photosynthesis, Gross primary productivity, Ecosystem respiration, Net ecosystem exchange, ERA5, MODIS, Sentinel-3, FLUXNET, Gaussian process regression, Global upscaling, Climate, Remote sensing, LAI, Vegetation products

## Abstract

Recent efforts in upscaling terrestrial carbon fluxes (TCFs) from eddy covariance (EC) flux towers have gained momentum with machine learning, capturing complex relationships between TCFs and their driving variables. We applied Gaussian process regression (GPR) models to upscale TCF products from tower-to-global scale and studied the predictive capacity of climate variables and leaf area index (LAI) across biomes (2004–2023). The developed GPR models (EC-GPR-TCFs) were trained with FLUXNET data, including gross primary productivity, ecosystem respiration, and net ecosystem exchange. LAI field measurements were combined with climate variables observed at EC towers, including soil variables at varying depths. Upscaling was realized in Google Earth Engine at varying spatial resolutions from 300 m to 5 km, employing the EC-GPR-TCFs models with the MCD15A3H-LAI product from MODIS, the S3-TOA-GPR-LAI product derived from Sentinel-3 (S3) and ERA5-Land climate data. Tower data availability for training and validating the model is reduced when combining multiple variables and measurements, a challenge addressed by the EC-GPR-TCFs models. Bidecadal temporal correlation analysis (2004–2023) between TCFs and predictor variables highlighted three key variables for estimating TCFs: LAI, shortwave incoming solar radiation (SW), and latent heat flux (LE), with the strongest correlations in forests of temperate and cold climates. Multi-year (2019–2023) intercomparison of EC-GPR-TCFs estimations against EC towers data for validation revealed consistent results with $R^{2}$ and rmse generally around 0.6 and below 3 μmol m^−2^ s^−1^, respectively. At the global scale, decadal (2010–2020) intercomparison against four benchmark TCF products (LPJ-GUESS, MOD17A2H, FLUXCOM, and SCOPE-GPR-TCFs) resulted in varying fits with corresponding median R and rmse values in the ranges 0.7–0.85; 1.97–4.36 μmol m^−2^ s^−1^ for GPP and 0.81–0.85; 2.20–3.38 μmol m^−2^ s^−1^ for RECO. Our analysis offers a deeper understanding of how climate predictors influence TCFs across biomes, providing a new perspective on TCF upscaling methodologies.

## Highlights

• GPR was used to upscale global terrestrial carbon fluxes (TCFs) from 2004 to 2023.
• TCFs upscaling revealed 20-year patterns in temperate, boreal, and crop ecosystems.
• LAI, LE, and shortwave radiation were key drivers of TCFs across biomes.
• Strong TCFs agreement showed Sentinel-3/5P data are effective proxy inputs.
• The upscaling method supports various satellite LAI products for mapping TCFs.

## 1 Introduction

According to the Copernicus climate service, the 2024 mean global temperatures exceeded the 1.5 °C limit above pre-industrial levels established in the Paris Agreement (https://climate.copernicus.eu/ accessed on 15th January 2025). Given the weight of the terrestrial carbon fluxes (TCFs) on the total carbon budget, and thus their role in mitigating the increase of temperatures (Keenan and Williams, 2018), accurate tools for quantifying TCFs are urgently needed to inform effective mitigation policies.

TCFs are driven by the opposing processes of photosynthesis and respiration. Vegetation incorporates CO_2_ from the atmosphere to synthesize organic matter, and during the inverse process, respiration, live organisms (including autotrophs and heterotrophs) emit CO_2_ to the atmosphere (Khadke et al., 2024). Photosynthesis rate is commonly expressed as gross primary productivity (GPP). Respiration, named RECO when referring to ecosystem respiration, is defined on similar terms (e.g., grams of carbon per day) in the reverse way (from the Biosphere to the Atmosphere). The difference between these two variables is referred to as the net ecosystem exchange (NEE) measured by eddy-covariance (EC) flux towers (Khadke et al., 2024, Baldocchi et al., 1988). Conceptually, NEE aggregated on a certain temporal scale (e.g., annual) corresponds with carbon stored, the same as the net ecosystem production (NEP), calculated from autotrophic carbon pools and heterotrophic carbon fluxes (Curtis et al., 2002). In reality, both NEE and NEP often diverge because of various measurement uncertainties (Bracho et al., 2012, Curtis et al., 2002). In this respect, the study of Curtis et al. (2002) points out the closest agreements of NEE with measurements of stored carbon based on biometric changes in two major pools, suggesting that, in practice, NEE is a more accurate metric of real carbon stored. Being the focus of this study, in the following, GPP, RECO, and NEE will be categorized as TCF products.

Inferring GPP from satellite data has for decades relied on the principles of light use efficiency (LUE) in photosynthesis, initially established by Monteith (1972). According to his principles, GPP can be quantified by determining empirical coefficients of LUE and the intercepting photosynthetically active radiation. From this theory, multiple algorithms have been developed for estimations of GPP through satellite-based spectral data, typically following similar approaches as the one defined in Running et al. (2015), in which GPP is calculated through the fraction of absorbed photosynthetically active radiation (FAPAR), retrieved from MODIS (Moderate Resolution Imaging Spectrometer) data, the usage of maximum LUE coefficients and the determination of environmental constraints (e.g., minimum temperatures and hydric stress) limiting real LUE.

Building upon the above fundamentals, physically-based land surface systems possess the versatility to simulate a diverse set of canopy fluxes (including carbon) by introducing parametrization of biophysical and biochemical processes (Haxeltine and Prentice, 1996, Farquhar et al., 1980). Some examples of these models include ORCHIDEE (Organizing Carbon and Hydrology in Dynamic Ecosystems) (Krinner et al., 2005), LPJ-GUESS (Lund-Potsdam-Jena General Ecosystem Simulator) (Martín Belda et al., 2022), SCOPE (Soil Canopy Observation of Photochemistry and Energy fluxes) (Tol et al., 2009), CliMA Land Wang et al. (2023), BESS (Breathing Earth System Simulator) (Ryu et al., 2011) and BEPS (Boreal Ecosystems Productivity Simulator) (Liu et al., 1997). These process-based systems are also currently being employed along with machine learning (ML) algorithms to provide efficient ways of assimilating products of terrestrial carbon fluxes, based on physical principles (e.g., hybrid models in Reyes-Muñoz et al., 2024, Girardin et al., 2008, Liu et al., 1997). Apart from physically-based models, also empirical data acquired through EC flux towers have been exploited to develop data-driven models. The FLUXCOM and the UFLUX initiatives (Songyan Zhu and Hill, 2024, Jung et al., 2020) are examples where ensembles of ML algorithms were developed to predict GPP and NEE based on MODIS reflectances and meteorological data.

The advantage of these data-driven models over purely physically-based models lies in their flexibility to exploit complex, often nonlinear, relationships between the variables used to predict TCFs, measured at the EC tower sites. In this context, Gaber et al., 2024a, Burton et al., 2023, Shangguan et al., 2023, Jung et al., 2020, Beer et al., 2010 and Zeng et al. (2020) explored models based on empirical relations between meteorological variables, vegetation properties, and TCFs. With the expansion of data and technical capabilities, research on the role of possible predictor variables is experiencing a surge, leading to a more nuanced understanding of these factors (Gaber et al., 2024a, Songyan Zhu and Hill, 2024). For instance, the role of soil variables, heat fluxes, and satellite vegetation proxies, including the potential for substituting some predictors with others, remains an active area of research (Reyes-Muñoz et al., 2024, Jonard et al., 2020, Camino et al., 2019). Particularly, recent works address the role of the soil temperature (TS), the soil water content (SWC), the solar-induced fluorescence (SIF) or leaf area index (LAI) as predictors of TCFs; e.g., the effect of soil temperature on parameters determining photosynthesis (such as the Rubisco carboxylation capacity - Vcmax, the electron transport rate, and the stomatal conductance) and therefore TCFs was demonstrated by Zhang and Dang (2005), while the SIF signal, the radiant flux re-emitted by vegetation between 650 and 800 nm, was also used to estimate Vcmax in Camino et al. (2019). The impact of SWC on the stomatal conductance and the SIF response to drought conditions was studied by Jonard et al. (2020). LAI is the main structural variable conditioning the coupling of ecophysiological processes; LAI corresponds with the one-sided green leaf area (broad-leaf canopies) or half of the total needle surface area (needle-leaf canopies) per unit ground surface area, influencing carbon, water, and energy fluxes (Baldocchi et al., 2002, Rhodes, 1971).

Yet, the usage of data-driven models is not without risk. The limitations of data-driven models typically come along with the availability of sufficient field representative data and a realistic balance for covering the present spatial diversity. In this regard, Gaussian process regression (GPR) models have been demonstrated to be among the most competitive ML algorithms for predicting biophysical variables, especially efficient when using small sample sizes for training global scope models (e.g., Reyes-Muñoz et al., 2024, Verrelst et al., 2015, Verrelst et al., 2012a, Verrelst et al., 2012b). Besides, of interest is that the GPR algorithm is designed within a Bayesian framework and provides uncertainties from posterior probability functions of the model (Rasmussen and Williams, 2006), allowing further understanding of the relation of predictor variables and target (Kovács et al., 2023b, Reyes-Muñoz et al., 2022, Verrelst et al., 2013).

These capabilities make GPR an attractive option when modeling TCFs. In comparison with alternative ML algorithms, e.g., neural networks (NN), GPR presents a simplified way to learn relationships without having to deal with decisions such as what architecture, what activation functions, or what learning rate should be applied, and the lack of a principled framework to answer these questions (Rasmussen and Williams, 2006). The probabilistic nature of GPR eases the search of adjusted hyperparameters automatically and produces full distributions over posterior predictions with the associated uncertainties, a notable advantage over competitive ML algorithms (e.g., NN, random forest). Recently, GPR has been successfully employed for predicting GPP and net primary production (NPP) on diverse combinations of environments (e.g., croplands, forests) through hybrid models trained with SCOPE simulations named as SCOPE-GPR-TCF in Reyes-Muñoz et al. (2024). Yet, that approach could not provide RECO and NEE as these variables are not included as SCOPE outputs. Also, the SCOPE-GPR-TCF models capitalized more on a diversity of satellite-based products that are considered proxies of environmental conditions, instead of incorporating a full set of climate variables.

Given all the above, this study aims to address the following questions: (1) whether data-driven GPR models trained with EC tower data from a combination of climatic variables and field-measured-LAI (named hereafter EC-GPR-TCFs models) produce consistent TCF products at tower-to-global scales, if they are consistent when being applied to distinct medium resolution satellite-based LAI products as derived from MODIS (500 m) and Sentinel3 (S3; 300 m) data streams and if the GPP, RECO, and NEE components estimated by EC-GPR-TCFs align with each other; (2) to compare the prediction strength of the predictors used in EC-GPR-TCFs models under varying biomes; and, (3) to explore whether the EC-GPR-TCFs models are spatiotemporally comparable with global benchmark TCF products derived from diverse methodologies, including machine learning, physical, and hybrid models. Those global TCF products use a wide range of variables, including vegetation information such as FAPAR or LAI and climate variables related to water and light availability such as evapotranspiration or soil temperature. The analysis will identify which methods yield comparable results despite their methodological differences and where improvements in accuracy, resolution, and processing speed can be achieved.

## 2 Methods

**Fig. 1:**
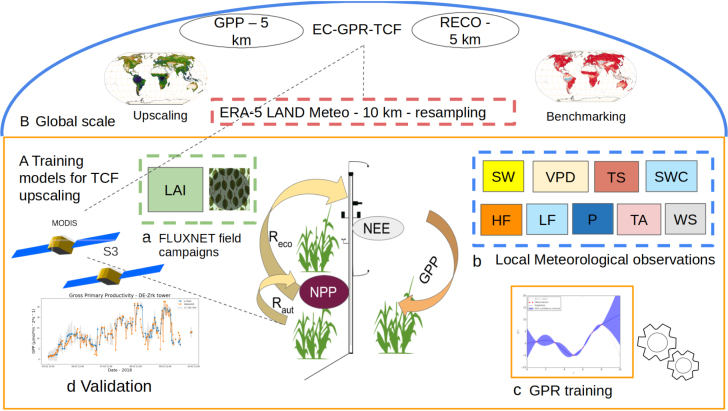
Conceptual methodological schema: (A) EC-GPR-TCFs models were trained with (a) LAI and (b) climate field observations, and subsequently validated against FLUXNET products at EC sites (d). (B) The EC-GPR-TCFs models were applied to MCD15A3H-LAI, S3-derived LAI and ERA5 data streams for upscaling of TCFs.

The workflow employed is illustrated in Fig. 1. Briefly, the steps followed for the TCFs estimating and upscaling can be summarized as follows: (1) collecting a tower-based data set including all the predictors (meteorology, LAI) and TCFs target variables (GPP, RECO, NEE); (2) tuning the GPR models; (3) implementation of the models in the Google Earth Engine (GEE) platform for global processing, and; (4) validation and spatiotemporal comparison against benchmark global TCF products. The following sections detail the steps taken.

### 2.1 GPR algorithm

We employed the GPR algorithm for the tower-based upscaling of TCFs, following the same approach as outlined in Reyes-Muñoz et al. (2024). The algorithm, based on a Bayesian framework, outputs a mean estimate $Y_{i}$ and a mean standard deviation $\sigma_{i}$ at new points, being $\sigma_{i}$ an indicator of the epistemic uncertainty provided by the model. Calculation of $Y_{i}$ is as: $Y_{i}=f\left(x_{i}\right)+\epsilon$, with $Y_{i}\in R$, and being ϵ an additive Gaussian noise with zero mean and variance $\sigma_{n}^{2}$, and $f\left(x_{i}\right)$ a Gaussian distributed random vector with zero-mean and a Covariance matrix K(x,x) quantifying sampling distances. A kernel function $k\left(x_{i},x_{j}\right)$ of type Asymmetric Square Exponential, also known as Radial Basis Function (RBF) (Camps-Valls et al., 2016) solves K(x,x), being defined as:

(1) $$k\left(x_{i},x_{j}\right)=\sigma_{s}^{2}\exp\left(-\frac{1}{2}\overset{D}{\underset{b=1}{\sum }}{\left[\frac{x_{i}\left(b\right)-x_{j}\left(b\right)}{\sigma_{b}}\right]}^{2}\right),$$

where $\sigma_{s}^{2}>0$ is the output variance while $\sigma_{b}$ is related to the relevance of dimension (or band) b in the prediction process: the higher $\sigma_{b}$, the lower informative content of b. The function $k\left(x_{i},x_{j}\right)$ is adjusted by maximizing $Y_{i}$ prediction likelihood over the function values f and a training matrix $X\in R^{D}$, as

$$\log p\left(y|X,\theta \right)=-\frac{1}{2}y^{T}{\left(K\left(X,X\right)+\sigma^{2}I\right)}^{-1}y-\frac{1}{2}\log\det\left(K\left(X,X\right)+\sigma^{2}I\right)-\frac{n}{2}\log2\pi$$

The maximization of the log marginal likelihood p(y|x,θ) provides the optimum value of the hyperparameters $\theta =\left\{\sigma_{s}^{2},\sigma_{1},\ldots ,\sigma_{D},\sigma_{n}^{2}\right\}$ to be used for K(x,x) estimation (Rasmussen and Williams, 2006).

Calculation of the $\sigma_{i}$ uncertainty for a given point is obtained as the standard deviation considering all possible posterior functions that fit the observed data:

(2) $$\sigma {}_{f}\left(x_{*}\right)=\sqrt{k\left(x_{⋆},x_{⋆}\right)-v^{T}v}$$

where $k(x_{⋆}$, $x_{⋆})$ indicates the kernel function calculated at the new input $x_{⋆}$; v a vector $v=L\setminus k_{⋆}$; L the low-triangular matrix calculated from the expression: $K+\sigma_{n}^{2}I_{N}$, with $\sigma {}_{n}{}^{2}$ the noise variance and $I_{N}$ the noise identity Matrix.

The RBF kernel is able to capture asymmetric responses often observed in biophysical parameters and TCFs derived from remote sensing data. TCFs exhibit different rates of change depending on external drivers (here bands), with gradual increases and abrupt declines due to stressors like droughts or temperature extremes. Unlike isotropic kernels, the RBF kernel accounts for these direction-dependent variations, improving predictive accuracy and uncertainty quantification. Additionally, it effectively handles non-stationary processes, making it well-suited for modeling spatiotemporal dynamics in ecological and climate studies. Furthermore, each sensor (MODIS, S3) has its specific observation characteristics, including varying spatial resolutions (500 m and 300 m), responding distinctly to mixed-pixel effects and spatiotemporal gaps. RBF kernels can model anisotropic correlations between spectral bands or across time, improving retrieval accuracy in noisy or incomplete datasets.

The practical implementation of the algorithm in a cloud computing infrastructure, such as GEE, is based on Reyes-Muñoz et al. (2022) and Pipia et al. (2021). See Rasmussen and Williams (2006) for details about the theory of GPR.

### 2.2 EC-GPR-TCFs models

To finetune the optimal hyperparameters of the EC-GPR-TCFs models, a matrix of X samples was collected from measurements recorded from EC flux towers included within the FLUXNET network (Pastorello et al., 2020) and distributed through the AMERIFLUX site at https://ameriflux.lbl.gov/data/flux-data-products/ and the ICOS site at https://www.icos-cp.eu/data-products (RI et al., 2024). The training data set combined data from meteorological variables and LAI obtained during field campaigns (see Fig. A.2). Methods for measuring LAI varied across ecosystem types, from hemispherical photography in forests to a combination of direct and indirect methods (e.g., ceptometers) in crop sites, Gielen et al. (2018). Depending on the site, the field measurements are referred either to real LAI, or to the surface area of all aboveground standing vegetation, including green as well as nonphotosynthetic parts (e.g., senescent material), named as plant area index (PAI), or with the photosynthetically active area of standing vegetation, named as green area index (GAI) (Gielen et al., 2018). For simplification, here we will treat all the vegetation measurement types as LAI.

**Fig. 2:**
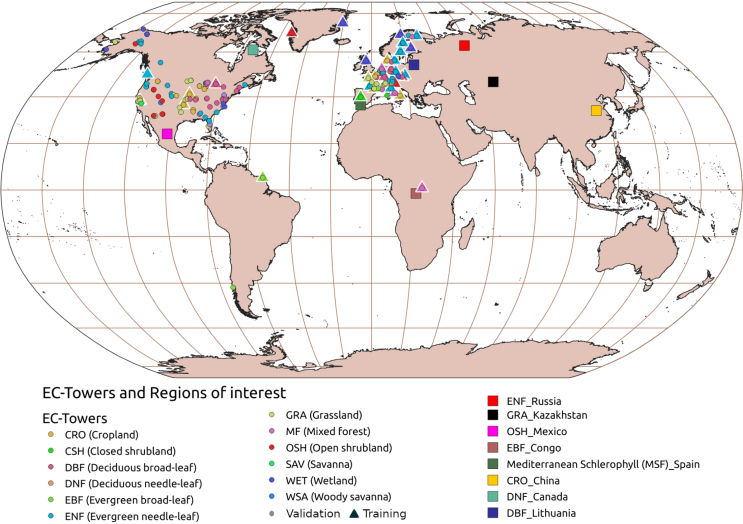
Location of the EC towers and geographical areas (squares) where the predictors-TCFs correlation analysis was applied. The selected geographical areas are distinctive of the main global biomes. The EC towers used for training the EC-GPR-TCFs models are represented as triangles, while the ones used for validation are shown as points.

A total of 1369 EC tower samples were collected with diverse meteorological variables and LAI field measurements on matching dates over the temporal period 2000–2024 (see Fig. 2 with the locations of the EC flux towers employed). The optimal models were consolidated after applying 10-folds cross-validation. Note that GPR models reported excellent local-to-global performances when trained with small sample sizes; see also Reyes-Muñoz et al., 2024, Kovács et al., 2023b, Estévez et al., 2022, Reyes-Muñoz et al., 2022, De Grave et al., 2020.

**Fig. A.1:**
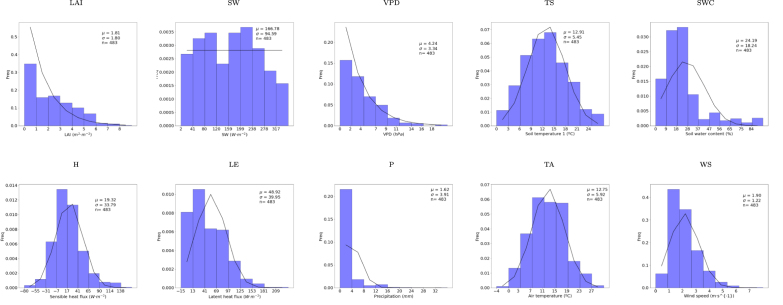
Histogram of values for the main predictors, obtained from the data set used for prediction and validation. Each variable is associated with a different probability density function.

The samples were collected over 9 vegetation types (see Fig. 2, Fig. A.3), existing nevertheless an imbalanced number of towers and samples for each vegetation type (see Fig. A.3). Croplands (CRO), evergreen needle-leaf (ENF), and deciduous broad-leaf forests (DBF), along with grasslands (GRA) and wetlands (WET), emerged as the most representative vegetation types, while evergreen broad-leaf forests (EBF), closed shrublands (CSH) and open shrublands (OSH) were the less representatives. The full set of variables used as predictors of the EC-GPR-TCFs models is shown in Table 1 along with the probability distribution function (see Fig. A.1 with the histogram plots for each variable). The available data fulfill a quality control threshold and are also gap-filled through the method MDS (Marginal Distribution Sampling) presented in Reichstein et al. (2005). The EC-GPR-TCFs models were trained with daily aggregated values to match the temporal aggregation of the input data, aiming to minimize errors arising from scale mismatches due to the nonlinearity between TCFs and the predictors (Reyes-Muñoz et al., 2024). The EC-GPR-TCFs models were trained in the machine learning regression algorithm (MLRA) toolbox of ARTMO (Automated Radiative Transfer Models Operator) (Verrelst et al., 2012c) and were subsequently exported into GEE by following the steps of Pipia et al. (2021) and coded in https://github.com/msalinero/ARTMOtoGEE (accessed on 8th July 2024).

**Table 1:** Predictor variables of the EC-GPR-TCFs models. The data were acquired from FLUXNET. The ranges shown here were used to train the models, as daily mean values (see Fig. A.1 and Fig. A.2 for more details). For flux variables, the negative is downwards and the positive is upwards.

Name	Label	Units	Range	Distribution
LAI	LAI	$m^{2}\;m^{-2}$	0–9.38	Exponential
Short wave incoming radiation	SW	${\text{W m}}^{-2}$	2.94–357.39	Uniform
Vapor pressure deficit	VPD	hPa	0.07–19.01	Exponential
Soil temperature (n depths, up to 3)	TS_n	°C	−0.79–28.02	Chi square
Soil water content (n depths, up to 3)	SWC_n	%	0–94.54	Gamma
Sensible heat flux	H	${\text{W m}}^{-2}$	−80.42–163.58	Chi square
Latent heat flux	LE	${\text{W m}}^{-2}$	−15.60–238.44	Gamma
Precipitation	P	mm	0–36.46	Exponential
Air temperature	TA	°C	−4.92–26.71	Chi square
Wind speed	WS	${\text{m s}}^{-2}$	0.64–8.41	Gamma

### 2.3 Data streams for inferring TCFs

The EC-GPR-TCFs models were subsequently applied to satellite data streams coming from the MODIS sensor (MCD15A3H-LAI), and OLCI (Ocean and Land Colour Instrument) on board S3 (S3-derived LAI), and the ERA5-Land reanalysis products (see Table 2). ERA5-Land was combined with either the MCD15A3H-LAI or the S3-derived-LAI product for validating the models and covering spatiotemporal dynamics at regional and global scales.

The MCD15A3H-LAI product (Myneni et al., 2015) relies on a Look-up Table (LUT) for inverting the Leaf Area Index (LAI), based on the spectral reflectance content in the red (648 nm) and near-infrared (NIR, 858 nm) bands. The LUT is generated from a 3D radiative transfer model (RTM). A backup algorithm uses empirical relationships between the Normalized Difference Vegetation Index (NDVI) and the canopy LAI and FAPAR. Inputs for the algorithm include vegetation structural type, sun-sensor geometry, bidirectional reflectance factors in the red and NIR spectral bands, and their uncertainties. The method involves comparing observed and modeled spectral reflectance across various canopy structures and soil patterns, representing a range of expected conditions for a given biome type. The standard MODIS C6 LAI/FAPAR products have a spatial resolution of 500 m and include LAI/FAPAR retrievals from MODIS Terra, Aqua, and a combined Terra+Aqua.

The S3-derived LAI product at a spatial resolution of 300 m has been developed in-house, relying on the same GPR algorithm used in this work (Reyes-Muñoz et al., 2022). The LAI product is retrieved directly from top-of-atmosphere (TOA) observations of the S3-OLCI instrument. Their retrievals are likewise obtained through a hybrid SCOPE-based GPR model that runs on S3-OLCI L1B radiance data in the spectral range between 400 nm to 1020 nm. By subsequently implementing the GPR model into GEE, these products proved to be globally applicable, and intra-annual comparison against related MODIS and Copernicus products evidenced its spatiotemporal consistency (Kovács et al., 2023b).

The ERA5-Land product (Muñoz-Sabater et al., 2021a), operated by the European Centre for Medium-Range Weather Forecasts (ECMWF), offers hourly information on surface variables. It is a refined version of the ERA5 climate reanalysis, focusing solely on land components with an improved spatial resolution of approximately 9 km grid spacing. The data set spans from 1950 to the present. ERA5-Land incorporates land surface hydrology within its model framework. This allows for a detailed representation of land processes and provides users with a consistent view of the decadal evolution of land variables. ERA5-Land also includes uncertainty information for all variables at reduced spatial and temporal resolutions, ensuring comprehensive data analysis.

**Table 2:** Overview of the MODIS and S3 vegetation product and the ERA5-Land reanalysis model used as real data-streams inputs (i.e., the eight predictor variables) for upscaling the EC-GPR-TCFs models.

Product	Predictor variables	Source	Spatial res.	Temporal res.	Method
MCD15A3H	**LAI**	MODIS	500 m	8-days	LUT inversion (3D RTM)
ERA5-Land	**SW, TS1, TS2, TS3, SWC1, SWC2, SWC3, H, LE, P, TA, WS**	ECMWF	0.1°	hourly - monthly	climate reanalysis
S3-GPR	**LAI**	S3	300 m	8-days	Hybrid model (SCOPE-GPR)

### 2.4 Upscaling of the TCFs models for global mapping

The EC-GPR-TCFs models were evaluated during the 20 years 2004–2023 across different spatiotemporal scales. First, at the tower scale, We estimated GPP, RECO and NEE over 8-days composites. These estimations were based on the satellite data streams (MODIS and S3-derived LAI) and ERA5-Land climate data which were also aggregated over 8-day periods for the temporal windows 2019–2023. The estimations were produced at the nominal spatial resolution of the satellites (500 m for MODIS and 300 m for S3-OLCI).

Second, a regional upscaling was conducted on central-west Europe based on S3 and ERA5-Land over a surface containing 11 EC towers belonging to 6 distinct vegetation types: CRO, DBF, ENF, GRA, CSH, and MF (see Fig. 2 with definitions). The same temporal windows 2019–2023 were selected to map mean annual values of TCFs over this region and study its spatial dynamics, based on the S3-derived LAI product at its nominal 300 m spatial resolution. To estimate TCFs at this spatial resolution, the ERA5-Land products were resampled through bilinear functions.

Third, we applied a 20-year global analysis through 8-day composites at a 5 km spatial resolution. Given that the input products (e.g., MCD15A3H-LAI and S3-derived LAI) and the MOD17A2H benchmarking product came at a temporal resolution of 8 days, we chose the 8-day format for uniform analysis.

### 2.5 Validation and benchmarking

The validation of the EC-GPR-TCFs models using EC tower data was conducted in two stages. First, 10-folds cross-validation was applied to evaluate the consistency of the GPR models during the training process, involving the iterative usage of 1232 samples for training and 136 for validation on the different folds, from the total collected 1369 samples (see Fig. 2). The cross-validation was based on daily estimates by using LAI field measurements and climatic variables observed at the EC towers. Second, several towers were used for multi-year (2019–2023) intercomparison across America and Europe for all the considered vegetation types. Fig. A.3 shows the details about the number of samples and towers used both for training and validation. For some vegetation types scarcely represented in the training (i.e. EBF, OSH, and CSH), we increased the number of samples for the validation in a second stage as field data were no longer limiting (i.e., satellite products were used for TCFs estimations). The training/validation distributions on the less represented vegetation types when the MCD15A3H-LAI product was used for the estimates were 17/616 (EBF), 38/887 (CSH), and 24/1703 (OSH). The reference products are available from AMERIFLUX and ICOS. GPP and RECO were obtained through partition methods (Lasslop et al., 2010), using a variable Ustar threshold to filter out data that may not be representative of ecosystem fluxes due to low turbulence conditions (Pastorello et al., 2020).

At a larger scale, the following products were prepared for intercomparison over the decade 2010–2020: FLUXCOM, LPJ-GUESS, and MOD17A2H as representative of empirical-ML, process-based, and LUE-semiempirical, respectively (see Table 3). We also included the SCOPE-GPR-TCFs products in the intercomparison analysis. The SCOPE-GPR-TCFs products developed in Reyes-Muñoz et al. (2024) are based on the synergy of S3 vegetation products and SIF retrieved from Sentinel-5P (S5P). These products come in various spatial and temporal resolutions: from daily to yearly, from 500 m to 0.5°. To enable calculating global intercomparisons, the EC-GPR-TCF products were resampled to match the resolutions of the intercompared products. In summary, the EC-GPR-TCF product was intercompared against FLUXCOM, LPJ-GUESS, MOD17A2H, and SCOPE-GPR-TCF representing a diversity of methods with different variables involved.

**Table 3:** Overview of the products used for benchmarking (GPP and RECO) against the EC-GPR-TCFs models.

Product name	Producer	Algorithm	Original resolution	Temporal resolution	Reference
FLUXCOM	Max Plank Institute for Biogeochemistry	Ensemble learning	5 km	Monthly	Jung et al. (2020)
LPJ-GUESS	Lund-Postdam-Jena	Process-based	0.5°	Monthly	Martín Belda et al. (2022)
MOD17A2H	EOSDIS LPDAAC	Empirical LUE	500 m	8-days	Running et al. (2015)
SCOPE-GPR-TCF	LEO - UV	GPR	300 m	8-days	Reyes-Muñoz et al. (2024)

### 2.6 Analyzing the relevance of predictors to estimate TCFs

To analyze the relevance of the selected predictors (see Table 1) in driving the TCF products, the GPR automatic relevance determination (ARD) covariance function was employed (Verrelst et al., 2016b). The constructed models involve an automated ranking of features, which were subsequently displayed in a polar plot following the method outlined by Ayala Izurieta et al. (2022) and further referred to as GPR-rank. The polar plot reveals the primary predictors influencing the data-driven models through the application of a scaling function, giving greater coefficients to predictors (or variables) of higher relevance. Also, Pearson and Spearman’s correlations were calculated between each of the predictors and the TCFs across the temporal range 2004–2023 over different analysis areas across the globe, shown in Fig. 2 and Table A.1. The Pearson correlation (R) measures the linear relationship between two variables, perfect for direct proportional relationships (Rodgers and Nicewander, 1988). Conversely, the Spearman correlation (ρ) assesses monotonic relationships, suitable when changes are consistent but not constant (Spearman, 2010). Both coefficients range from −1 to +1, indicating the strength and direction of the association. Pearson’s R is a complete measure of association only if the joint distribution is bivariate normal. Spearman’s ρ is robust to outliers and does not require a linear relationship, making it appropriate for non-normally distributed data (Schober et al., 2018, Khamis, 2008, Fowler, 1987).

## 3 Results

### 3.1 Validation

**Fig. 3:**
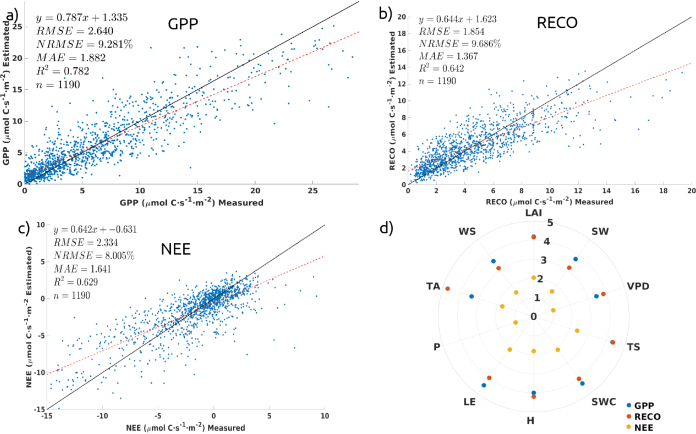
Cross-correlation validation (10-folds) results with scatterplots showing EC-GPR-TCFs estimations against observations at EC towers for GPP (a), RECO (b), and NEE (c). A polar plot in (d) shows the predictors GPR-Ranking, with higher scores towards the edge of the circle.

The reliability of the trained EC-GPR-TCF models was evaluated through 10-fold cross-validation, resulting in total coefficients of determinations $R^{2}$ of 0.78, 0.64, and 0.63 respectively for GPP, RECO, and NEE respectively (Fig. 3). The nrmse ranged between 8% (NEE) and 9.69% (RECO).

**Fig. 4:**
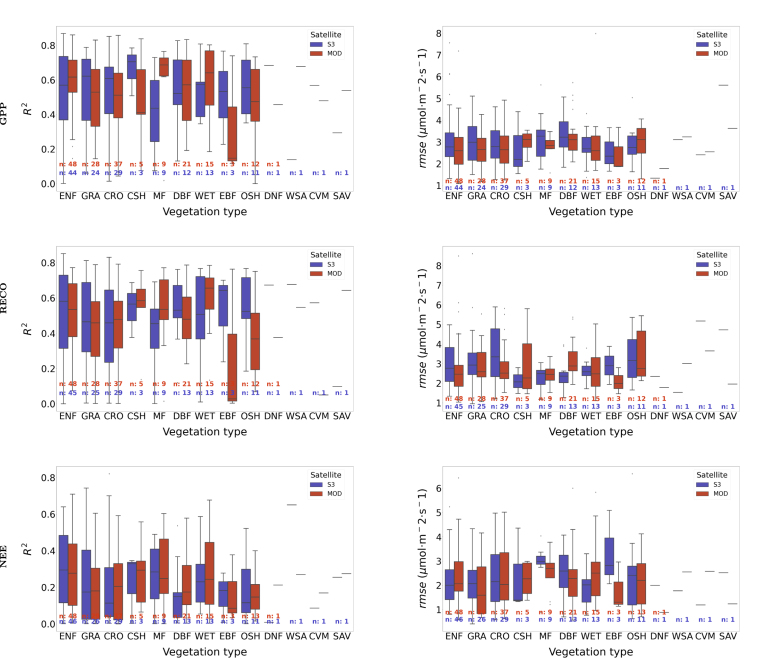
Validation of the EC-GPR-TCFs models against EC towers observations during 2019–2023. In blue, results obtained for EC-GPR-TCF models applied on S3-derived LAI (300 m) and in red on MCD15A3H-LAI (500 m). (For interpretation of the references to color in this figure legend, the reader is referred to the web version of this article.)

Next, to assess the performance of the EC-GPR-TCFs models, we applied them to global satellite and ERA5-Land datasets from 2019 to 2023. We then compared the model outputs with the products available from FLUXNET EC towers located in America and Europe (Fig. 4, Fig. A.4).

The plots in Fig. 4 contain boxplots with the $R^{2}$ and rmse metrics obtained on the EC towers and classified by vegetation type and satellite input (blue for S3-OLCI and red for MODIS). The obtained medians of $R^{2}$ and rmse ranged respectively for GPP and RECO between 0.4 and 0.8, and between 1.5 and 3 μmol m^−2^ s^−1^. Considering both metrics and the number of EC towers used by vegetation type, the most consistent performances were found to be associated with ENF, GRA, CRO, DBF, WET, and OSH, with medians of $R^{2}$ around 0.6 and of rmse around 3 μmol m^−2^ s^−1^. On EBF, the metrics indicated the highest accuracy, with $R^{2}$ around 0.8 and rmse around 1.5 μmol m^−2^ s^−1^ (GPP), when the EC-GPR-TCF models were employed on S3-OLCI. However, only three EC towers were available for validation on these sites, limiting therefore the analysis of spatiotemporal validation over EBF ecosystems. The $R^{2}$ metrics for GPP were superior when using S3-derived LAI compared to MCD15A3H-LAI for most vegetation types, except for MF and WET. The largest performance difference between S3-derived LAI and MCD15A3H-LAI occurred for EBF. Also in terms of rmse using S3-derived LAI estimations excelled for the majority of vegetation types. For RECO, similar patterns were encountered, and also for NEE with generalized under-performance.

### 3.2 Tower-to-global upscaling

**Fig. 5:**
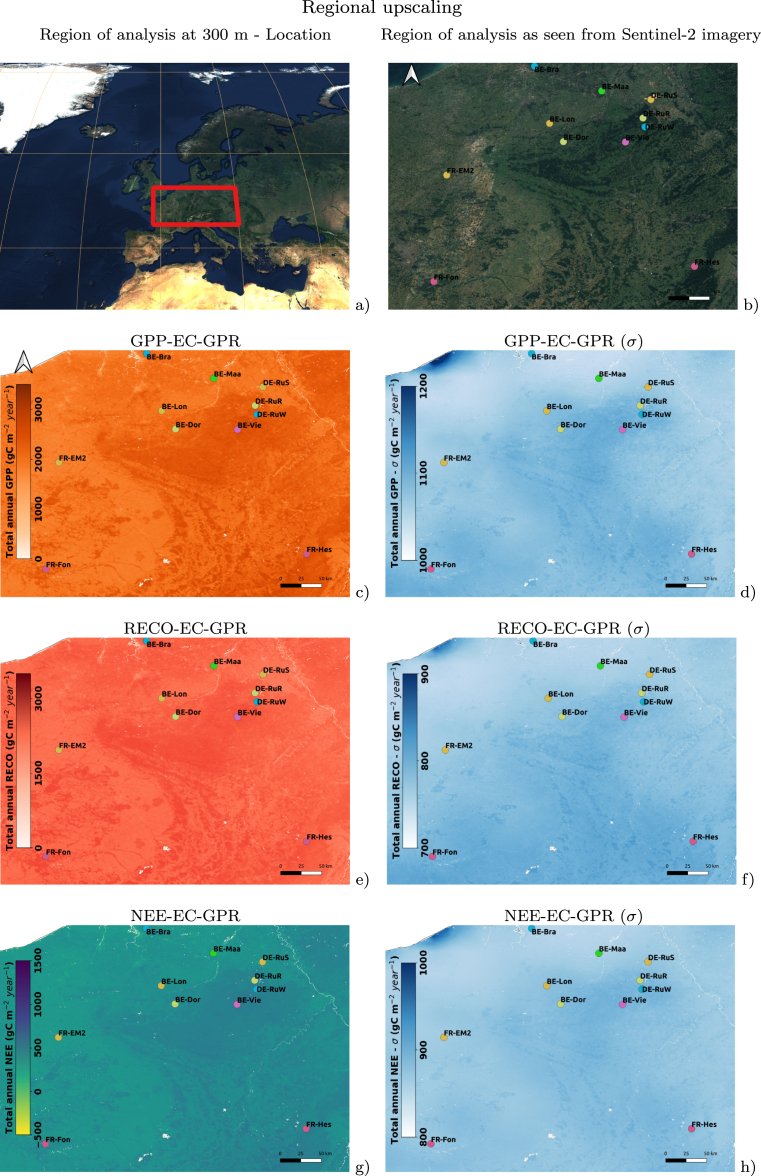
From up to bottom: Maps of GPP (c), RECO (e), and NEE (g) mean annual values (2019–2023) over a surface containing 11 EC flux towers belonging to 6 different categories of vegetation type (ENF, CSH, CRO, GRA, MF and DBF). The uncertainties (σ) maps are also displayed in blue color at the right column (d, f, and h). (For interpretation of the references to color in this figure legend, the reader is referred to the web version of this article.)

After the validation of the EC-GPR-TCFs models against EC tower data, a regional map of TCFs covering EC-towers belonging to CRO, DBF, ENF, GRA, CSH, and MF are shown in Fig. 5 at a spatial resolution of 300 m. The maps were produced by employing the EC-GPR-TCF models to S3-OLCI and ERA5-Land. By rows, estimates and uncertainties (σ) of mean annual GPP, RECO, and NEE are displayed. The values correspond to total mean quantities calculated over 2019–2023. The maps highlight a spatial distribution driven by the vegetation types, with the forests reaching the maximum values of TCFs: 2800 gC m^−2^ y^−1^ (GPP), 1900 gC m^−2^ y^−1^ (RECO) and 900 gC m^−2^ y^−1^ (NEE), and the CRO and CSH associated to lower values: 1900 gC m^−2^ y^−1^ (GPP), 1700 gC m^−2^ y^−1^ (RECO) and 400 gC m^−2^ y^−1^ (NEE). The epistemic uncertainties, quantified through the σ layer, followed a smooth spatial distribution with patterns similar to the estimates, i.e., maximum values were found associated with maximum estimate values. In general, the σ layer represents uncertainties around 30%–40% of the estimates, being proportionally higher for NEE, with values ranging between 50% up to more than 100% of the NEE estimates.

**Fig. 6:**
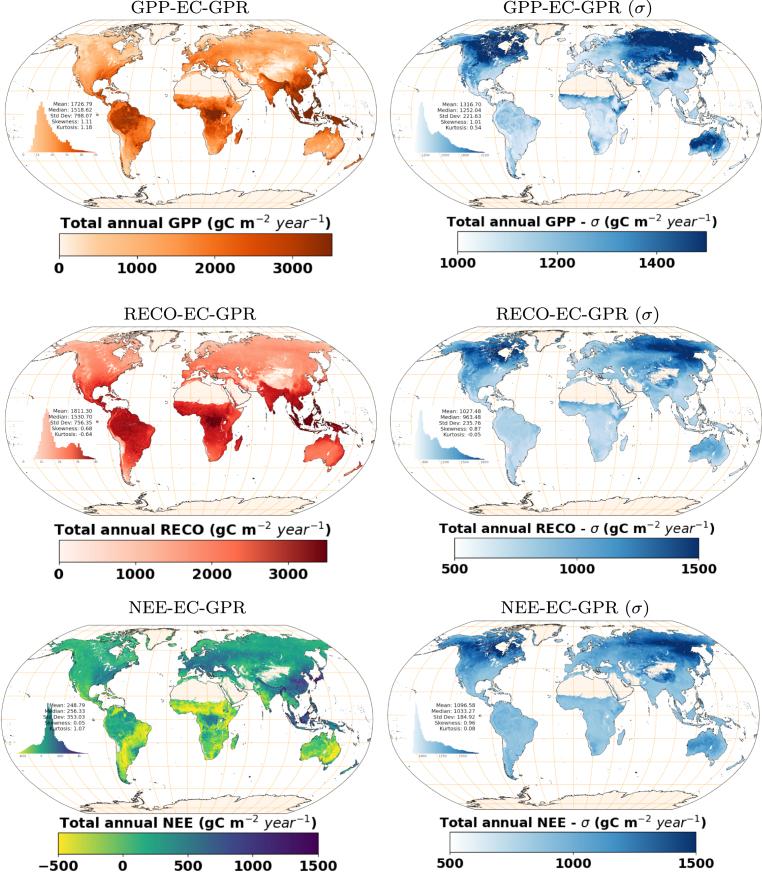
Total annual quantities of GPP, RECO and NEE obtained for the entire series 2004–2023 by applying EC-GPR-TCFs on MCD15A3H-LAI and ERA5-Land. NEE positive values are referred to fluxes in the direction from atmosphere to biosphere.

Following, the potential of the EC-GPR-TCFs models for global mapping is shown in Fig. 6. TCFs maps were produced by employing the EC-GPR-TCFs models to the MCD15A3H-LAI and ERA5-Land products at a spatial resolution of 5 km. The accumulated annual mean values for 2004–2023 show an expected spatial distribution highlighting Earth’s biomes. The highest values of GPP, RECO, and NEE occur in tropical areas, presumably linked to the availability of light, water, and dense vegetation (i.e., high LAI). Along the equator, the increase in GPP is slightly greater than that of RECO, resulting in high NEE values that indicate a persistent carbon sink over time. Similar patterns are observed in temperate forests (e.g., in Europe). The σ layer presents the highest uncertainty values on higher latitudes (around 60°Northward). In the south, and especially over surfaces subjected to Atlantic influence, uncertainties are smoother and TCFs spatial patterns vary linked to land cover type, with higher values exposed over forest areas (e.g. tropical forests).

### 3.3 Predictors relevance: GPR ranking and analysis of temporal correlation with TCFs

**Fig. 7:**
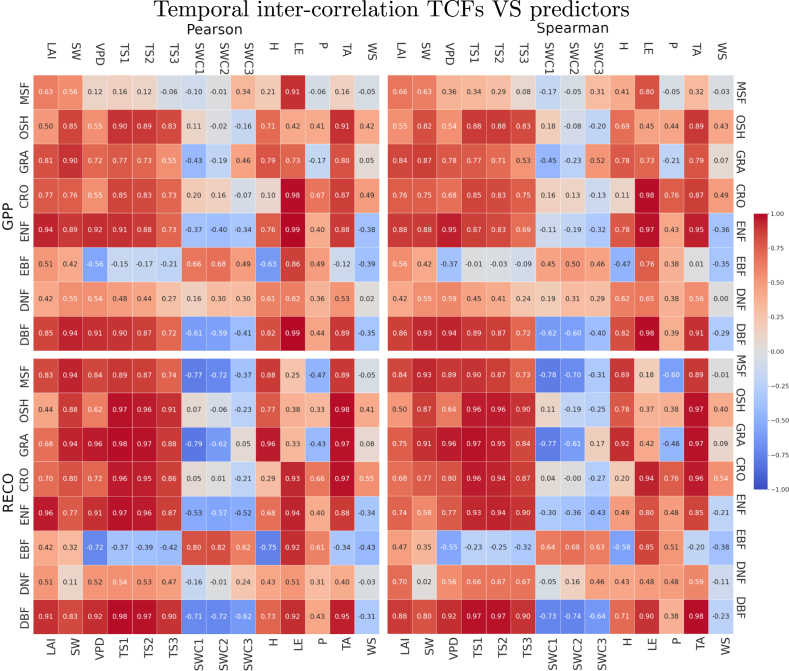
Heat maps indicating Pearson and Spearman temporal correlation (2004–2023) of GPP and RECO with predictor variables (MCD15A3H-LAI and ERA5-LAND) on surfaces for analysis globally distributed.

The predictors’ relevance in driving the TCF products is assessed both at the tower and global scales. The first analysis is addressed from the data set used for training the EC-GPR-TCFs models and visualized in the polar plot (Fig. 3). Next, the global analysis is performed from the time series of TCFs and predictors (Fig. A.6) and represented as “heat maps” (Fig. 7).

The polar plot of the GPR input variables indicates the most influential predictors in the EC-GPR-TCF models (Fig. 3). For GPP and RECO, although the relevance coefficients are nearly similar for most of the predictors, LAI, LE, and the soil variables (TS and SWC) present slightly higher relevance for estimating GPP, meanwhile for RECO, TA highlights among other variables by its highest relevance. In both cases, P appears as a minor predictor. The relevance coefficients of the NEE predictors indicate that these variables play a smaller role in the NEE model as opposed to GPP and RECO, with LAI, TS, SWC, and LE occupying the highest positions of the ranking. Notably, GPR ranking identified LAI, SWC, TS, and LE as strongly influencing both GPP and RECO, while SW, LE, and WS primarily affected GPP, and TA and VPD primarily affected RECO.

The temporal dimension was also analyzed to capture the biome-specific temporal behavior of the TCFs (see Fig. 2 and Table A.1), and also to understand the role of the input predictors in driving the temporal dynamics. Fig. A.6 shows the time series of GPP and RECO across 20 years along with the predictors. The annual seasonality of GPP and RECO is well represented for croplands (CRO-China), DNF forests (DNF-Canada), and also for DBF (DBF-Lithuania). In contrast, for tropical (EBF-Congo) or Mediterranean forests (MSF-Spain), intra-annual oscillation is more unpredictable.

The temporal correlation analysis (Pearson and Spearman) in the heat maps (Fig. 7) reveals that LAI, SW and LE are the most relevant variables with high correlation in most of the vegetation types (e.g. GPP-LE Pearson correlation ranges between 0.61 and 1.00 and RECO-LE between 0.42 and 0.95). The relevance of SWC as a predictor of GPP and RECO is nevertheless not so evident despite the GPR-Rank results in Fig. 3. For instance, in the DBF area (DBF-Lithuania) correlations are negative. This region is limited by other factors during winter, such as light (SW) or temperatures (TA, TS), which at the same time determine the phenology of this ecosystem (Baldocchi et al., 2018, Froelich et al., 2015). Furthermore, the statistical properties of the time series (e.g. variance, seasonality, etc.) constrain the degree of correlation between variables. Thus, SWC strongly determines TCFs across a wide range (see Fig. 3). Still, these correlations may be weaker for specific climates, such as those analyzed in the DNF-Canada CRO-China and OSH-Mexico, with constrained variation ranges (see Fig. 7). The heat maps of Fig. 7 also reveal how areas linked with high seasonality correlate better with the predictors and vice versa for low seasonality. For example, evergreen forests, such as the tropical EBF-Congo and the Mediterranean MSF-Spain, correlate only strongly with LE, LAI, SW, and SWC.

### 3.4 Global benchmarking

**Fig. 8:**
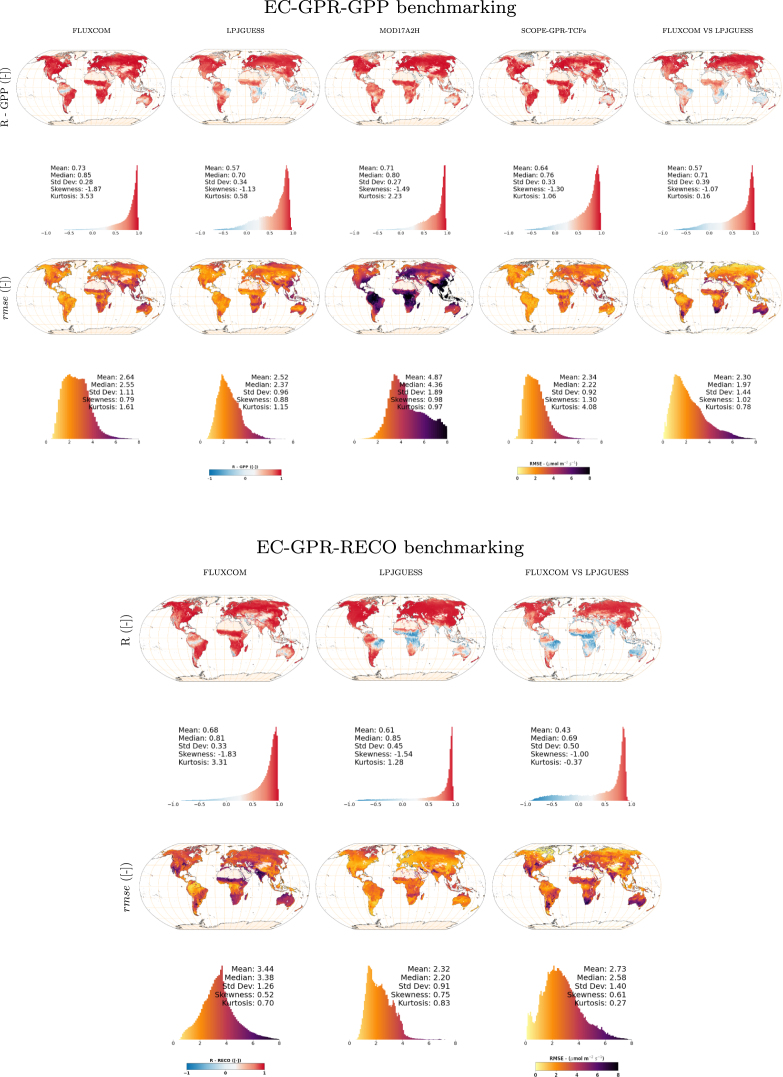
R and rmse intercomparison maps: EC-GPR-TCFs (GPP and RECO) estimations (2010–2020) against FLUXCOM, LPJ-GUESS, MOD17A2H and SCOPE-GPR-TCF products.

Building upon the spatiotemporal analysis of the EC-GPR-TCF product in previous sections, a decadal (2010–2020) intercomparison assessment against benchmark, gridded, global products is provided next. Global GPP and RECO EC-GPR-TCFs outcomes are intercompared with estimations of FLUXCOM, LPJ-GUESS, MOD17A2H, and SCOPE-GPR-TCF. Fig. 8 summarizes the intercomparison analysis through global maps of the statistics R (Pearson) and rmse. The key findings of the intercomparison results, extracted by data pairs, are given below:

(1) EC-GPR-TCFs VS. FLUXCOM

The intercomparison against FLUXCOM data stream reveals a generally high correlation, except in the case of tropical forests and sparse vegetation north of India and Patagonia. Higher rmse values were encountered over areas composed of tropical forests and herbaceous vegetation and shrublands, mainly in the African continent, Southeast of Asia, and northern latitudes, with common mean values around 4 μmol m^−2^ s^−1^. Peaks of more of 6 μmol m^−2^ s^−1^ were found in India for RECO.

(2) EC-GPR-TCFs VS. LPJ-GUESS

The intercomparison against the LPJ-GUESS data stream resembles the previous case, although here the correlation worsened mainly in the Southern hemisphere, but the rmse marked smoother differences on those regions linked with the highest differences in the previous case (EC-GPR-TCFs Vs. FLUXCOM), especially for RECO.

(3) EC-GPR-TCFs VS. MOD17A2H

The intercomparison against the MOD17A2H product resulted in a similar R correlation as with the FLUXCOM GPP product, except in the tropical forests of Amazon and Indonesia where the intercomparison improved, and the shrublands around Argentina where it worsened. However, in terms of rmse, the intercomparison reached the highest differences with regards to the other analyzed cases, with peaks of 8 μmol m^−2^ s^−1^. The RECO variable was discarded in this analysis as it was unavailable from the set of MODIS-based products.

(4) EC-GPR-TCFs VS. SCOPE-GPR-TCFs

The intercomparison against the SCOPE-GPR-TCFs data stream provided a good balance in terms of R and rmse with a median correlation around the globe of 0.76 and rmse of 2.2 μmol m^−2^ s^−1^.

(5) FLUXCOM VS. LPJ-GUESS

The intercomparison between FLUXCOM and LPJ-GUESS is also provided as an independent reference. The results are comparable with cases (1) or (2), with generally low correlations (both for GPP and RECO) in the southern hemisphere but with higher rmse values in the South of Africa and Australia (herbaceous vegetation), and shrublands in the East of the USA and Mexico. The rmse maps show nevertheless lower differences in central Africa and Indonesia than in other cases, probably indicating an overestimation of the EC-GPR-TCFs models in these regions.

## 4 Discussion

This study offers novel insights into modeling TCF products using data-driven GPR models. The models are trained on EC tower data and then upscaled globally using satellite data. Driven by climate variables (ERA5-Land) and LAI observations (derived from MODIS and S3), the GPR models yield promising results for operational TCFs monitoring providing a viable alternative to existing benchmark products such as FLUXCOM, LPJ-GUESS, MOD17A2H, and SCOPE-GPR-TCF.

Despite the presentation of a diverse range of ML algorithms in previous studies to upscale TCFs from EC tower data (e.g., Songyan Zhu and Hill, 2024, Jung et al., 2020, Jonard et al., 2020, Zeng et al., 2020, Beer et al., 2010), so far the GPR algorithm has limited prior applications in TCF mapping. For instance, Camps-Valls et al. (2015) explored GPR for ranking drivers of TCFs, and more recently, Campos-Taberner et al. (2024) estimated global TCF products through multioutput GPR guided by MODIS time series. This study presents an additional perspective with a diverse set of variables, including information on the soil (TS, SWC) and LAI information across biomes. Moreover, in contrast to those previous GPR studies, here a detailed multi-year intercomparison analysis against benchmark products was conducted. A discussion about the intercomparison results, the importance of the TCFs predictors involved, and the advantages and limitations of the presented EC-GPR-TCFs models is addressed next:

### 4.1 Consistency across scales and between TCFs variables

The tower-to-global upscaling results highlighted the following points:

First, the ecosystem diversity appeared well represented across scales, with a global picture reflecting the contribution of the main biomes of the Earth as primary producers as well as carbon sinks (NEE). The global spatial distribution of annual accumulated net ecosystem exchange (carbon stored) roughly corresponds with Nelson et al. (2024) with the highest quantities identified in the tropical regions of Africa, America and Indonesia. The global maps of total annual quantities estimations of EC-GPR-NEE reflect similar spatial patterns, coinciding also over Australia, the Sahel or arid regions in India and around the Caspian Sea as the minor contributors of annual NEE, and boreal forests as medium contributors. As also observed by Nelson et al. (2024), EC-GPR-TCFs estimate higher carbon sink values over temperate forests and croplands in middle latitudes than reported in Campos-Taberner et al. (2024) and Jung et al. (2020). Furthermore, the EC-GPR-NEE models neglect lateral transfers of carbon due to factors such as deforestation or fires, with a high impact in total carbon net deficits according to Pan et al. (2011) over the entire southern tropical regions (deficits indeed point out these regions as net sources of C over 1990–2007 according to this work). These factors should be incorporated within new-generation EC-GPR-NEE models to enable estimating total carbon net deficits. Additionally, at a local-to-regional scale (e.g., see maps in Fig. 5), the analysis showed that spatial patterns are mostly shaped by the change in the land cover type.

Second, the consistency between the estimated TCFs is assessed based on the reliability of the partitioned GPP and RECO fluxes. In this regard, we compared NEE calculated as the difference between GPP and RECO from corresponding EC-GPR-TCFs models (see Fig. A.5) against NEE retrieved from the EC-GPR-NEE model. Both results showed a similar spatial distribution of NEE. However, values in the tropics emerged more prominently from the EC-GPR-NEE model, while NEE calculated in the tropics by differences between GPP and RECO resulted in a smoother gradient towards adjacent regions. The analysis reveals that EC-GPR-RECO produces estimations proportionally higher in these areas, with a latitudinal gradient of RECO close to the one linked to GPP. Conversely, the EC-GPR-NEE model appears more realistic, meaning that the EC-GPR-RECO model throws overestimated values in the tropics. The calibration of the EC-GPR-RECO model could be significantly enhanced by increasing data availability in tropical regions.

### 4.2 Analysis of the EC-GPR-TCFs uncertainties

The provided epistemic uncertainties reveal that the EC-GPR-TCFs models behaved less precisely in northern latitudes and in certain scarce vegetated areas in the southern hemisphere (e.g. Australia in the case of GPP). Limitations of the MODIS-LAI product to optimally capture seasonality in boreal forests, as described in Heiskanen et al., 2012, Yang et al., 2006, are reflected in the uncertainties analysis. This is primarily due to the impact of snow contamination and high solar zenith angles on LAI retrieval, mostly in the northern latitudes. Complementary tests in Fig. A.7 showed the impact of these factors in the increase of uncertainty. In contrast, the models produce more trustful values on temperate forests and even in the tropics. The spatiotemporal dynamics of the epistemic uncertainties are influenced by the degree of similarity between the predictor input ranges and those used during the model training. Thus, the smooth uncertainties found in the tropics mean that the models were trained on similar ranges. Nevertheless, tropical regions exhibit complex characteristics, including lush evergreen forests, intricate vertical vegetation structures, and relatively stable environmental conditions with minimal seasonal variations in day length. Besides, only three EC towers are available for data collection within tropical EBF. All these factors, combined with the nonlinear relationship between TCFs and their driving variables, may limit the ability of the EC-GPR-TCFs models to capture the dynamic behavior of TCFs in the tropics accurately. Yet encouragingly, the intercomparison analysis between EC-GPR-TCFs and the FLUXNET products improved substantially when using the S3-derived LAI (300 m) instead of the MCD15A3H-LAI (500 m) product for estimating TCFs on these three sites.

As for the propagation of other errors, the TCFs estimations may be affected by inaccuracies in the ERA5-Land data streams, as reported in da Silva et al., 2024, Gomis-Cebolla et al., 2023 and Yang et al. (2006). Additional tests in Fig. A.8 revealed bias of ERA5-Land key variables for estimating TCFs when intercompared with measurements at EC sites, highlighting those of LE at EBF leading to potential overestimations of TCFs, as well as those of P at CSH sites linked with underestimations. Importantly, in those tropical forests affected by rapid land use changes, ERA5-Land tends to underestimate increasing trends in TA and P (da Silva et al., 2024), causing potential underestimation of TCFs over time given the positive correlation between these variables and TCFs. Furthermore, in arid or semi-arid regions, overestimations of P in summer (Gomis-Cebolla et al., 2023) can also have an impact on TCFs estimations due to its link with other predictors highly correlated with TCFs such as LE, VPD, or SWC. The impacts of high SW variances in these areas for the estimation of TCFs may also be important. At the global scale, the studies of Martens et al. (2020) and Muñoz-Sabater et al. (2021a) highlight a generalized overestimation of LE, involving a potential overestimation of TCFs given the strong positive correlation between LE and TCFs.

Finally, uncertainties related to temporal variability and the use of 8-day aggregation scales also impact the results, owing to the non-linear relationship between weather conditions and TCFs. The significance of temporal sampling is especially evident during extreme weather, where climate variability intensifies (Chai et al., 2025, Li et al., 2021, Pan et al., 2020, Frank et al., 2015). Drastic changes in temperature and precipitation anomalies, as well as pathogen pest outbreaks, alter vegetation physiology, which can further propagate to TCFs variations (Frank et al., 2015, Reichstein et al., 2013). Also, extreme events such as frosts, floods, heavy storms, and fires can cause direct damage to vegetation and pose indirect impacts through land–soil modification. Ultimately, long droughts are presumed to have the strongest and most widespread effects on TCFs at a global scale, especially in forests, given their large carbon stocks (Frank et al., 2015, Reichstein et al., 2013).

### 4.3 Validation and benchmarking

The tower-scale intercomparison results in Section 3.1 revealed an overall moderate match with the FLUXNET data observed at the EC sites in the case of GPP and RECO. Detailed analysis on those EC towers reporting good correlations for GPP and RECO reveals a common pattern: the land surface appeared as uniformly distributed over wide spatial extensions. For example, the estimates at towers coded as US-xST, US-xDC, US-BZs, and US-BZo, located on DBF, GRA, ENF, and WET respectively, presented $R^{2}$ values over 0.8 and rmse below 2 μmol m^−2^ s^−1^. Conversely, the EC towers reporting lower correlations (e.g., US-xSB, US-Rpf, IS-ICh) are often associated with transition areas or surfaces containing multiple subcategories of land uses (e.g., landscape fragmentation and heterogeneity) within the same footprints, adding more complexity to the EC-GPR-TCFs models to produce their estimations. In these cases, the input ranges may be unknown by the models, or cause biases due to the grid cell heterogeneity and the nonlinear relation between TCFs and their predictors.

The validation against EC towers was generally more accurate when employing EC-GPR-TCFs models on S3-derived LAI as opposed to using MCD15A3H-LAI. The S3 enhanced spatial resolution (300 m) and the number of bands for capturing surface spectra along the visible and near-infrared turns out advantageous for inferring TCFs. This is encouraging, considering that new-generation satellites are being developed to ensure the continuity of the S3 missions whereas MODIS exceeded its lifetime.

The inferior performance observed for NEE can also be attributed to the varying measurement footprints of EC towers unmatched by the predictor data-streams grid cells used in the EC-GPR-NEE estimations. As described in Chu et al. (2021), varying measurement heights, underlying vegetation, and ground surface characteristics as well as wind directions and atmospheric turbulence provoke changes in EC measurements footprints in the range between $10^{3}$ and $10^{7}$ ${\text{m}}^{2}$. Footprint variability greatly impacts the NEE intercomparison where the EC-GPR-NEE modeled outputs are compared against EC measurements. Contrarily, the GPP, and RECO were intercompared against partition models relying on meteorological variables where changes in their footprint measurements may be weaker. Similar NEE intercomparison results were found by Nelson et al. (2024) on aggregated temporal scales. Yet, when using shorter temporal scales (i.e., hour) the results drastically improved, suggesting that modeling NEE on lower temporal scales leads to improved results, as also confirmed by Jung et al. (2020).

The decadal intercomparison against the global benchmark TCF products resulted in varying spatiotemporal patterns. Notably, the results obtained against the SCOPE-GPR-TCFs models (Reyes-Muñoz et al., 2024) excelled with top benchmark metrics. In this case, the algorithm was the same but the variables and the data streams differed (MODIS&ERA5-Land vs. S3&S5P). The intercomparison results against SCOPE-GPR-TCFs suggest that the diversity of meteorological variables employed in the EC-GPR-TCFs models could be replaced by the synergy of other variables (S3-derived vegetation products and TROPOMI-SIF), coinciding with what was analyzed by Songyan Zhu and Hill (2024) regarding the substitution of satellite-based vegetation products predictors of TCFs by a complete set of meteorological variables, or the opposite way.

The potential substitution of predictor variables (e.g., temperature, LE, or SWC replaced by satellite-based SIF and FAPAR) to estimate TCFs was especially verified in boreal and temperate forests with good intercomparison metrics between EC-GPR-TCFs Vs. SCOPE-GPR-TCFs. Even in the Amazon forests, the intercomparison between these two products presented lower differences than when using other benchmark products (i.e., LPJ-GUESS, FLUXCOM, or MOD17A2H). In CRO, the intercomparison results (EC-GPR-TCFs Vs. SCOPE-GPR-TCFs) presented moderate to good differences metrics. However, in shrublands and herbaceous vegetation areas EC-GPR-TCFs and SCOPE-GPR-TCFs estimates did not correlate well. The photosynthesis mechanisms of shrub/herbaceous vegetation (including C4, CAM photosynthesis pathway), commonly adapted to dry conditions, pose a challenge when it comes to disentangle the relationships between the employed variables in both models, and therefore further work is needed to address these relationships. In this regard, the upcoming FLEX (FLuorescence EXplorer) mission designed for capturing SIF at an unprecedented spatial resolution of 300 m (Drusch et al., 2016) is expected to unlock new possibilities in the development of TCF products by better capturing the photosynthetic activity as well as a better understanding of the mechanisms between photosynthesis and climate, especially in vegetation stress conditions (Van Wittenberghe et al., 2021, Jonard et al., 2020). The intercomparison yielded the poorest correlation against the LPJ-GUESS product in the Southern hemisphere, especially apparent in the case of the RECO intercomparison with a vast part of the African territory linked with negative correlations.

Divergences in the products intercomparisons can be due to the following factors: as a dynamic global vegetation model (DGVM), LPJ-GUESS considers plant functional types, biochemistry of the soil, and ecosystem dynamics through complex parametrization (Martín Belda et al., 2022). The other products (FLUXCOM, MOD17A2H, and EC-GPR-TCFs) rely upon empirical relationships learned in diverse local conditions. The poor correlations that emerged in the Southern Hemisphere can be attributed to the low representativeness of these specific ecosystems formed by a diversity of shrublands and grasslands (Olson et al., 2001). EC flux towers are significantly less prevalent in the Southern Hemisphere than in the Northern Hemisphere (Songyan Zhu and Hill, 2024, Pastorello et al., 2020, Hill et al., 2017). Furthermore, the spatial resolutions of the MODIS-based or S3-based LAI products capture better the landscape heterogeneity than the coarse-resolution LPJ-GUESS model (at 0.5°), causing another source of mismatch. However, in terms of rmse, the intercomparison against LPJ-GUESS resulted in lower differences as opposed to MOD17A2H (GPP) and FLUXCOM (RECO). The significant discrepancies observed between EC-GPR-GPP and MOD17A2H can be attributed to several factors. These include: (1) the use of a fixed maximum LUE in MOD17A2H, which might not be optimal for all conditions, (2) inaccuracies in land cover classification, and (3) errors propagated by FAPAR inputs, as detailed in Wang et al. (2017) and Tagesson et al. (2017). The deviations observed between EC-GPR-RECO and FLUXCOM-RECO may be related to how the RECO values are derived from the NEE measurements and the above-discussed footprint mismatches between modeled and measured NEE. Altogether, the degree of similarity between TCF products is mostly driven by the method employed, the spatiotemporal resolution of the input data streams, and the variables used as predictors.

### 4.4 Relevance of the used predictors across biomes

The analysis of the TCFs predictors relevance, i.e., ranking results of EC-GPR-TCFs predictors in Fig. 3 and bidecadal temporal correlation of the TCFs with predictors in Fig. 7, revealed three key variables with strong relevance in the estimation of TCFs over all the analyzed biomes: LAI, SW, and LE. LAI determines the structure of the canopy and is fundamental for quantifying terrestrial carbon and energy fluxes (Parker, 2020, Xie et al., 2022). SW represents the incoming energy used by vegetation to produce photosynthesis. LE corresponds with the energy associated with the evapotranspiration process occurring during the photosynthesis (Cicuéndez et al., 2023) and follows similar global spatiotemporal patterns of GPP and RECO (Jung et al., 2011). Other driving variables with implications across biomes are addressed next:

In arid environments (e.g., OSH-Mexico, GRA-Kazakhstan), SWC did not correlate well against GPP and RECO. In OSH-Mexico, the ecosystems are mainly composed of xerophyte vegetation, adapted to dry conditions, and the soil top layers hardly retain water, explaining therefore the existing low correlation between SWC and GPP/RECO. In this area, SW along with soil and air temperatures are the major drivers of TCFs. In GRA-Kazakhstan, a change in correlation between SWC and GPP/RECO from negative to positive as the SWC layer becomes deeper may be related to the ability of steppe grasslands species to produce photosynthesis with limited water availability and the deep root systems to access water from deeper soil layers (Li et al., 2023).

In semi-arid environments such as MSF-Spain, only LE, LAI, and SW present significant correlations with GPP, with LE as the main driver. The evergreen sclerophyll vegetation present on these ecosystems is adapted to a dry season in summer, tolerating shortage of water and high temperatures. However, the correlation of temperatures with RECO was positive, possibly linked to higher metabolic activity of vegetation.

In the analyzed croplands in CRO-China, similarly to previous cases, SW along with TS and TA explained most of the variability of GPP/NPP. Nevertheless, here P and LE gained major importance with regard to previous cases, meanwhile, H correlated worst, highlighting that these crops (mainly wheat, corn, and rice) are more dependent on irrigation. Interestingly, WS also presented a moderate correlation with GPP/NPP that may be associated with changes in temperatures and/or precipitation patterns.

Both in DBF-Lithuania and ENF-Russia, both surfaces belonging to snow-humid climates (according to the classification in Kottek et al. (2006)), the correlations against GPP/RECO presented similar schemes, with weak correlations between P and GPP/RECO and negative correlations between SWC and GPP/RECO. In these areas, solar energy and variables directly linked with it (i.e. TS, TA) are the limiting factors for GPP/RECO.

In the Tundra (DNF-Canada), predictors correlations against GPP /RECO were only moderate to low, with H and LE being the strongest drivers. The area, characterized by a cold climate, is mainly composed of moss and lichen with tight intra-annual variations of GPP and RECO (see A.6). This combination explains the lower intra-annual correlations. Nevertheless, an inter-annual decrease in GPP was observed over the temporal range 2004–2023, probably due to a response of the existing vegetation to a warming inter-year process.

Finally, EBF-Congo conditions are linked to a steady climate characterized by constant warm temperatures, day length, and the abundance of water. There, the main drivers are LE, SWC, LAI, and P by order of importance.

Along with the identified drivers, VPD was also found an important driver of GPP/RECO temporal dynamics in most cases, being widely investigated as a limiting factor in dryness conditions (He et al., 2021, Yuan et al., 2019, Goodrich et al., 2015). Yet, its relative contribution is difficult to disentangle (Middleby et al., 2024), given the significant correlation between VPD and TA (TA is an explanatory variable of VPD). Generally, TCFs-VPD correlations were found mostly positive except for EBF-Congo (GPP and RECO). A similar negative correlation across six tropical EBF species was found between VPD and three productivity parameters (net photosynthesis, stomatal conductance, and water use efficiency) when increasing VPD above 0.7 KPa (see Middleby et al. (2024) for more details). Also, the correlation between VPD and GPP in CRO-China, OSH-Mexico, and MSF-Spain was weak, possibly due to the adaptation of vegetation to dry conditions.

P correlation with GPP or RECO also varied with the biome type with a maximal correlation in CRO-China and tropical (EBF-Congo) regions, coinciding with the findings of Yuan et al. (2021). A weaker link was observed in the other biomes. According to Khadke et al. (2024), P causal link with NEE or RECO is not immediate but probably implies a delayed response of vegetation through soil moisture. Also, P (along with clouds) influences SW indirectly driving TCFs dynamics.

The bidecadal temporal correlation analysis identified significant predictors linked with TCFs across biomes. Regarding the soil variables, the temporal correlation did not show significant differences in predictors at the three soil depths except in GRA-Kazakhstan. As a follow-up analysis, causal relationships between these predictors and TCFs can be established, e.g., through Granger causality analysis, as demonstrated in Kovács et al. (2023a) and Cicuéndez et al. (2023).

### 4.5 Advantages and limitations of the EC-GPR-TCFs models

The application of the EC-GPR-TCFs models present the opportunity to improve the global upscaling of TCF products directly from canopy-level tower observations with models implicitly calibrated with field data. This approach improves current efforts in upscaling with models trained with samples of satellite-based vegetation products (Campos-Taberner et al., 2024, Gaber et al., 2024a), avoiding a source of error due to scale mismatch between point-to-pixel observations as well as possible biases due to the assumptions of the underlying retrieval algorithms. Along this line, the EC-GPR-TCFs models, trained without biases associated with specific satellite-based products, are designed to be used on any variant of LAI satellite-based product (e.g., MODIS, S3) and at any spatial resolution (Stoy and Quaife, 2015). At the same time, the introduction of a high processed-level biophysical variable (LAI), as opposed to lower processed-level products such as reflectances in Jung et al. (2020) or derived spectral products, e.g., vegetation indices (e.g., Pei et al., 2022, Dong et al., 2015) allows a more concise understanding of the role of the variables involved in the production of TCFs. Whereas LAI is considered an essential climate variable (Neilson and Drapek, 1998) that can be quantified in the field and is a driver of photosynthesis (e.g., Bonan, 1993), reflectances are driven by a broader set of land features, such as soil composition or land cover type, and other biophysical variables (e.g., FAPAR), making determining the unique influence of each studied variable more challenging.

Another advantage of using GPR as the core algorithm is that GPR models can lead to accurate performances with small input training data sizes (e.g., Estévez et al., 2022, Berger et al., 2021, De Grave et al., 2020, Verrelst et al., 2016a, Camps-Valls et al., 2016). Regarding the role of training data, Songyan Zhu and Hill (2024) points out the availability of representative data as a dominant factor for training ML models consistent on different ecosystem types, as opposed to the limited impact of the algorithm or the environmental drivers. The GEE platform provided a suitable environment for the application of the EC-GPR-TCFs models, with a wide database (including MODIS, S3, and ERA5 products) connected with a powerful infrastructure for processing data through parallel computation services (Gorelick et al., 2017). GPR’s ability to excel even with limited training data makes it a powerful tool for overcoming data scarcity challenges. The trained lightweight GPR models make fast and seamless processing into GEE possible with an entire global collection over 20 years of 8-days-steps (spatial resolution of 5 km) mapped in 35 h (file size of 1.36 Gb per variable). At the same time, GPR proves advantageous for TCF prediction when incorporating numerous predictors. GPR’s ability to handle small sample sizes effectively allows for leveraging a richer set of (often limited available) features, potentially improving model performance. Moreover, on the output side, GPR provides an epistemic uncertainty interval, so enabling to track per-pixel the fidelity of the model (García-Soria et al., 2024, Verrelst et al., 2013).

Regarding the nature of the input data, the optimization of the training data sets to meet realistic conditions is more straightforward than in the case of using hybrid models, where typically the training data sets are produced on representative ranges obtained from database compilations (e.g., Reyes-Muñoz et al., 2024, Estévez et al., 2022, De Grave et al., 2020) and possible combinations are more uncertain, leading to suboptimal models. Although several studies address the problem of optimization of physically-based hybrid models in different ways (e.g., Berger et al., 2021, Camps-Valls et al., 2018, Verrelst et al., 2016a), data-driven models offer the opportunity to explore a realistic combination of predictor variables for inferring TCF products more directly.

Amongst the encountered limitations of the EC-GPR-TCFs models, it should be noted that the information to build the EC-GPR-TCFs models is restricted to what is locally measured, affecting both the global representativeness of data and the variables employed. We remark on the lack of samples on EBF for training the models. When field data is absent, an alternative to address this limitation may be the addition of synthetic data to the training data set, as pursued in Reyes-Muñoz et al. (2024). Regarding the limitations of the selected variables, the employed field data set lacked sufficient data on FAPAR and SIF, preventing their incorporation into the EC-GPR-TCFs models. FAPAR is long and widely recognized as a crucial input variable for modeling (Monteith, 1972), fundamental in LUE-based models (Running et al., 2015). SIF also has a strong connection with GPP (Guo et al., 2024, Beauclaire et al., 2024). Here, incorporating LAI, which reflects the potential for leaf canopy interception of SW, in conjunction with other climatic factors (e.g., SW, TS, or SWC) yielded valuable insights into the dynamics of TCFs.

The limitations of the proposed approach further include the simplification of distinct vegetation density measurement types (GAI, PAI, and LAI) into a single category (LAI) for training the EC-GPR-TCFs models. This assumption may have a different impact on the TCFs estimations across the global biomes. In forests, PAI is a more descriptive variable of the total vegetation surface, and is therefore measured at the EC towers located on these sites (Gielen et al., 2018). The inclusion of components of vegetation as stems or branches during the PAI measurements involves that the EC-GPR-TCFs models are trained with total values of vegetation surface (including photosynthetically and non photosynthetically surface) higher than expected for its equivalent LAI on the same trees. Although there is evidence that for some species, components other than leaves also contribute to photosynthesis (Chen et al., 2023), here the TCFs estimated by the EC-GPR-TCFs models may be underestimated due to the usage of LAI data streams, instead of PAI. Meanwhile, in grasslands, the more suitable variable for describing total vegetation surface aboveground is GAI, with stems accounting for total photosynthetic material (Ávila-Lovera et al., 2020). Similarly as in the forest areas, in grasslands, we may encounter underestimations of TCFs as stems are not captured by the LAI data streams. These underestimations may be especially pronounced for stressful conditions, where the stems increase their proportional contribution to canopy carbon gains compared with leaves (Tokarz et al., 2021, Nilsen, 1995). Although further studies are needed to quantify the global bias in TCF estimations caused globally by neglecting these vegetation components, as a reference, Braghiere et al. (2019) and Chen et al. (2012) focused on the impact of neglecting foliage clumping in estimating global GPP from LAI, an issue also related with the importance of describing fully the canopy structure. Braghiere et al. (2019) and Chen et al. (2012) quantified global underestimations of GPP when using effective satellite-based LAI products, instead of clumping-corrected LAI, with the highest differences on the tropics up to −100 g C m^−2^ y^−1^ and −500 g C m^−2^ y${}^{-}1$ (Chen et al., 2012). Furthermore, Li et al. (2024) also quantified the impact of the foliage clumping on regulating land surface heat fluxes with decreases of 65% in H and increases of 2% in LE, both variables found correlated with TCFs in this work.

Another important question to consider is the quantification of CO_2_ fertilization effect, playing also a critical role in realistic estimations of TCFs (Jung et al., 2020), especially for detecting long-term trends. Although EC-GPR-TCFs do not include CO_2_ as input for estimating TCFs, an improved version of the presented models may also rely on this variable for dedicated studies on the role of atmospheric CO_2_ concentration on TCFs estimations across vegetation types.

Finally, regarding the availability of tower data, the low representativeness of the extreme temperature values (e.g., values below 0 °C or upper 30 °C) in the data set used for training the EC-GPR-TCFs models likely limits the EC-GPR-TCFs models to respond to those temperatures leading to vegetation stress or even absence of photosynthesis (Bhattacharya, 2022, Moore et al., 2021).

## 5 Conclusions

The growing need for global TCFs estimation methods coupled with limited field data for model calibration and validation necessitates optimizing how these models utilize existing data. In this context, here we presented a GPR data-driven method to optimize models of TCF products with EC tower observations. The developed models, named EC-GPR-TCFs, were then implemented on MCD15A3H-LAI, S3-derived LAI and ERA5-Land data streams using GEE to upscale and apply the TCF products globally. The EC-GPR-TCFs models were subsequently intercompared against the FLUXCOM, LPJ-GUESS, MOD17A2H, and SCOPE-GPR-TCF products across 2010–2020. Both the validation and the intercomparison analysis, as well as the examination of the target variables and how they are aligned, revealed generally consistent patterns across scales. Special cases are observed in tropical evergreen forests and arid or semiarid regions in the Southern Hemisphere, with a generalized mismatch between all the intercompared products. The temporal correlation analysis between predictors and TCFs identified LAI, SW and LE as key drivers strongly predictive in all the biomes. Other relevant predictors were linked with water availability (SWC, VPD, and P) or with incident solar energy (TA, TS, H), acting as limiting factors over OSH-Mexico (water availability) and DBF-Lithuania (light availability). Summarizing, our work consolidated that:

• The presented workflow can be adapted to upscale TCFs across varying spatiotemporal scales, with field-data-trained models transferable to satellite data.
• The performance of the EC-GPR-TCFs models proves the capacity of lightweight GPR models trained with small sample sizes to quantify TCF products consistently, contributing towards optimization of data usage for data-driven models.
• Our analysis suggest that some of the EC-GPR-TCFs predictors, such as H, LE, or soil variables, could potentially be substituted by a combination of satellite-derived vegetation products. Our findings demonstrate that using a combination of LCC, LAI, FAPAR, FVC, and SIF from S3 and S5P data in the SCOPE-GPR-TCF model yielded results comparable to those achieved by EC-GPR-TCFs. This suggests that the synergistic use of S3 and S5P data can effectively capture the influence of key climatic variables.
• The satellite spatial resolution plays a key role in better capturing the global dynamics of TCFs, with overall superior validation results when employing S3-derived LAI at a nominal spatial resolution of 300 m as opposed to MCD15A3H-LAI at 500 m.

Altogether, these findings reinforce the effectiveness of data-driven GPR models for tower-to-global upscaling TCF products, particularly successful for analyzing TCFs on local conditions and notable for extrapolation at a regional-to-global scale, e.g., as such processing is easily possible within GEE. Eventually, the EC-GPR-TCFs models can be further tailored when new data sources become available at EC towers, e.g., SIF and FAPAR observations.

**Fig. A.2:**
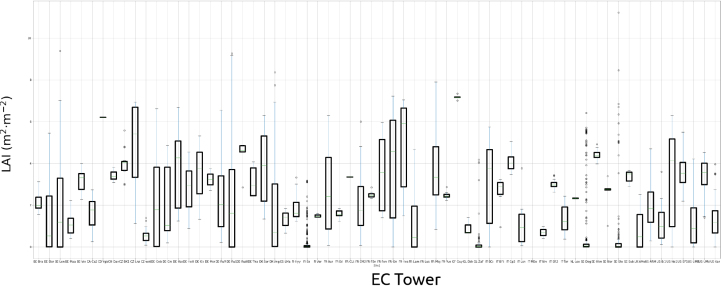
Boxplots with LAI measurements on field campaigns (time range 2000–2024) around EC tower locations, used for training EC-GPR-TCFs, provided by ICOS and Ameriflux.

## CRediT authorship contribution statement

**Pablo Reyes-Muñoz:** Writing – original draft, Visualization, Validation, Software, Resources, Project administration, Methodology, Investigation, Formal analysis, Data curation, Conceptualization. **Dávid D.Kovács:** Writing – review & editing, Visualization, Software, Resources. **Jochem Verrelst:** Writing – review & editing, Writing – original draft, Supervision, Project administration, Methodology, Investigation, Funding acquisition, Conceptualization.

## Declaration of competing interest

The authors declare that they have no known competing financial interests or personal relationships that could have appeared to influence the work reported in this paper.

## Figures and Tables

**Fig. A.3:**
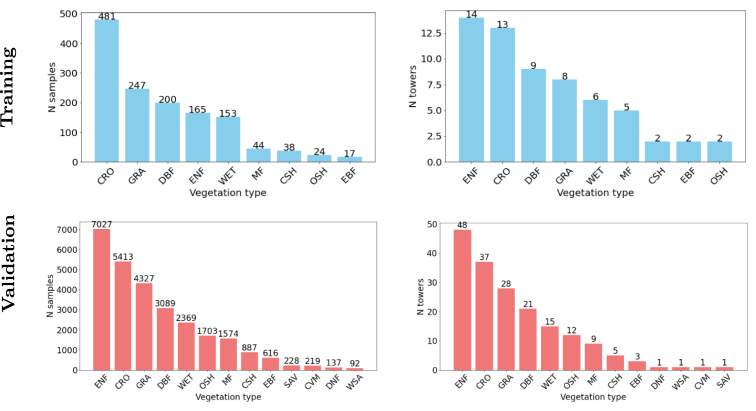
Histogram of number of samples and towers representative of each land cover type, for training (first row) and for final validation at EC towers (second row).

**Fig. A.4:**
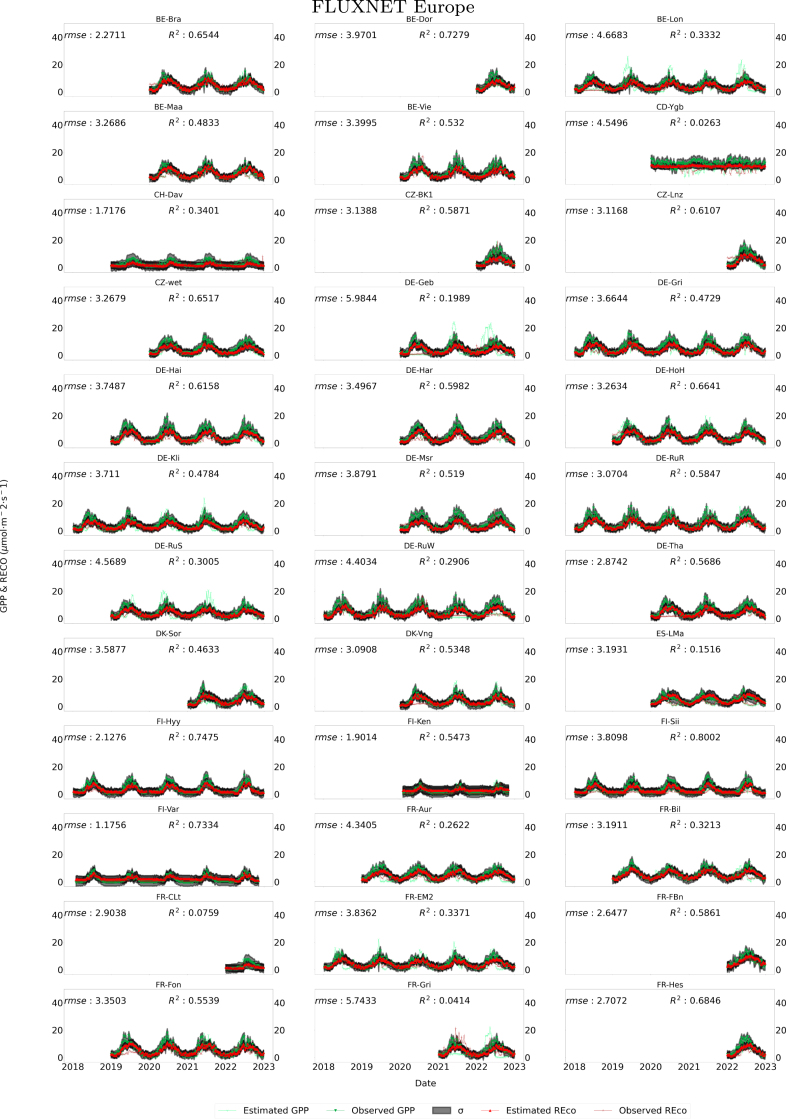

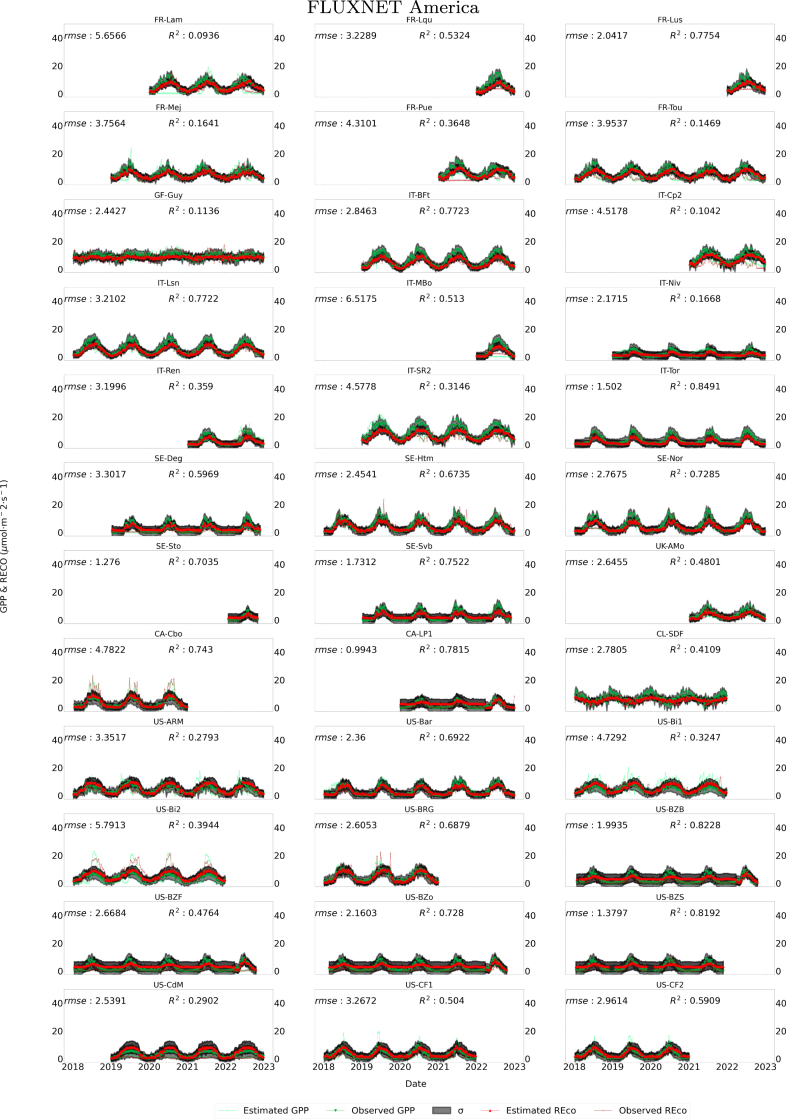

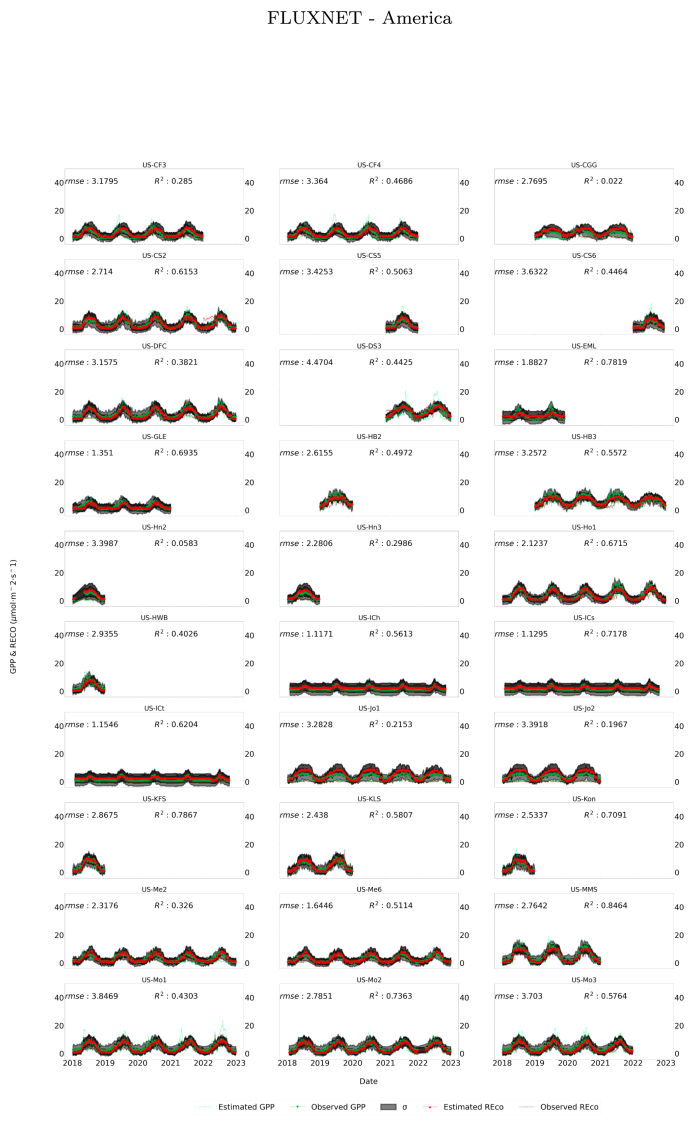
Intercomparison of GPP and RECO estimations at 108 flux tower sites over Europe and America, on 8-days temporal aggregation scale.

**Fig. A.5:**
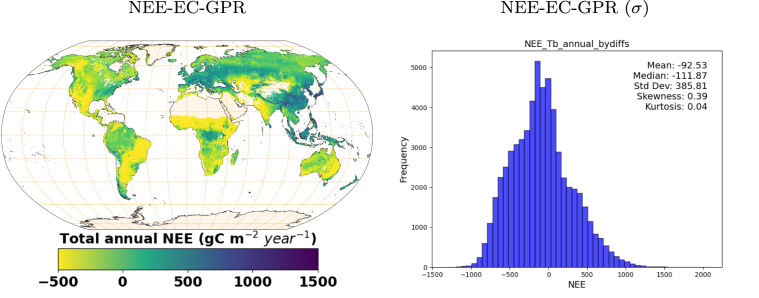
Total annual quantities of NEE obtained by differences between EC-GPR-GPP and EC-GPR-RECO estimations for 2004–2023.

**Table A.1:** Selected areas for temporal correlation analysis between TCFs and predictors, identified through the “Copernicus Global Land Cover Layers”. Biome classification based on Olson and Dinerstein (1998).

Land cover	Extension (ha)	Biome
Evergreen needle-leaf forest (ENF)	3,666,086.67	Taiga
Evergreen broadleaf forest (EBF)	2,153,746.44	Tropical rainforest
Deciduous needle-leaf forest (DNF)	407,769.25	Tundra
Deciduous broadleaf forest (DBF)	36,364.56	Temperate forest
Mediterranean sclerophyll forest (MSF)	84,159.79	Mediterranean forests, woodlands and scrub
Cropland (CRO)	84,159.79	Grassland (GRA)
Shrubland (SHR)	168,621.02	Grassland
Grassland	429,633.84	Grassland

**Fig. A.6:**
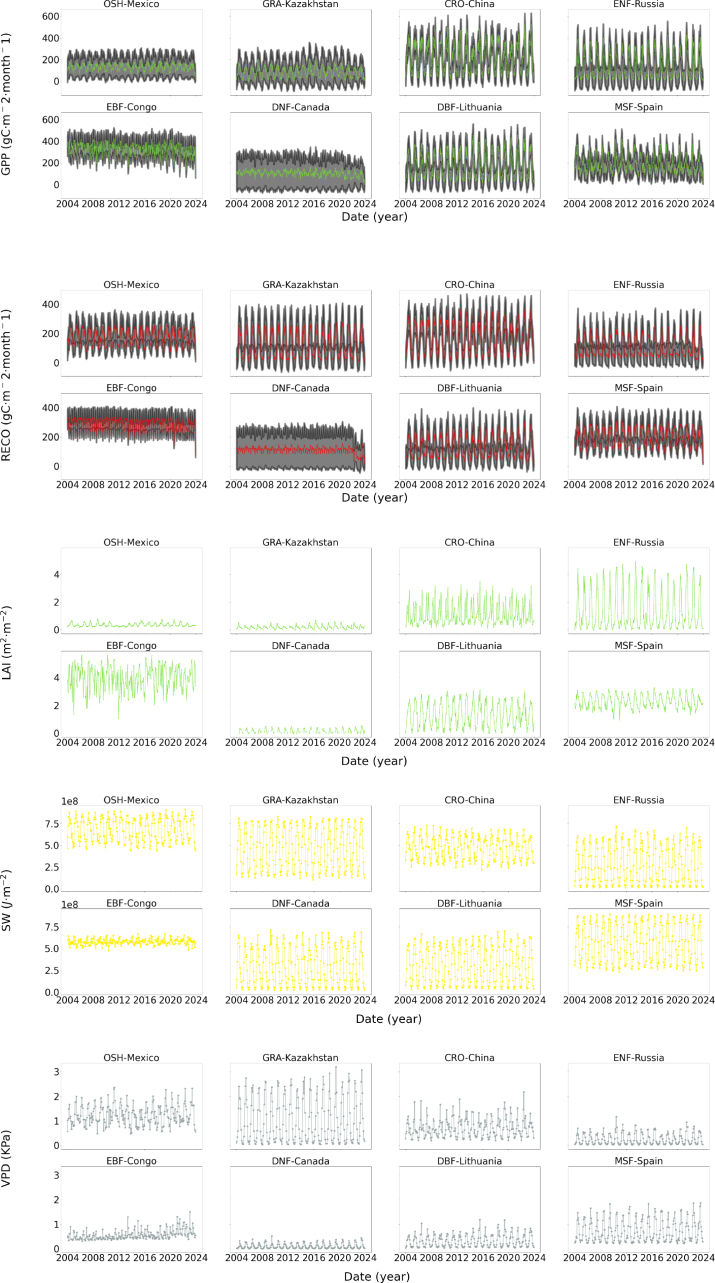

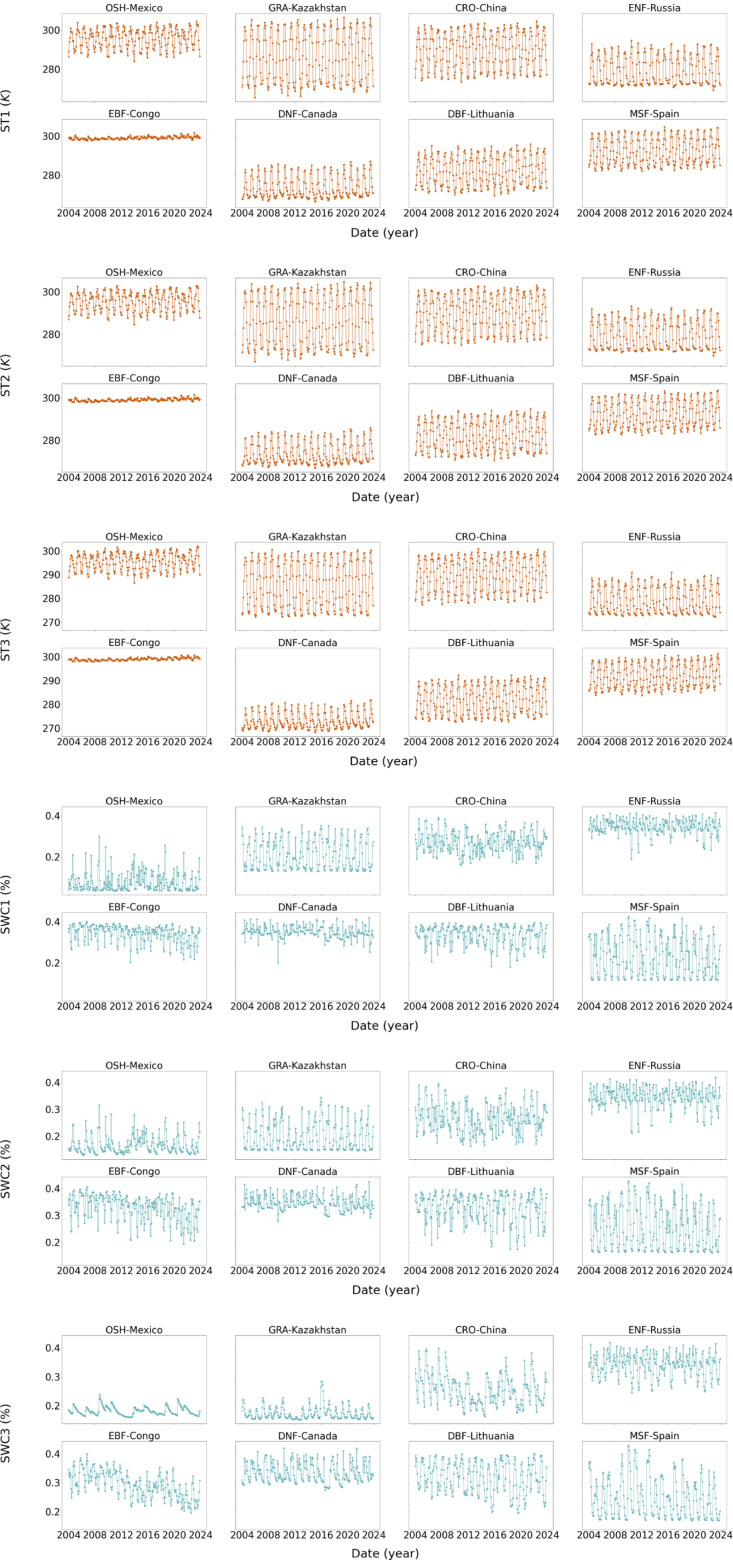

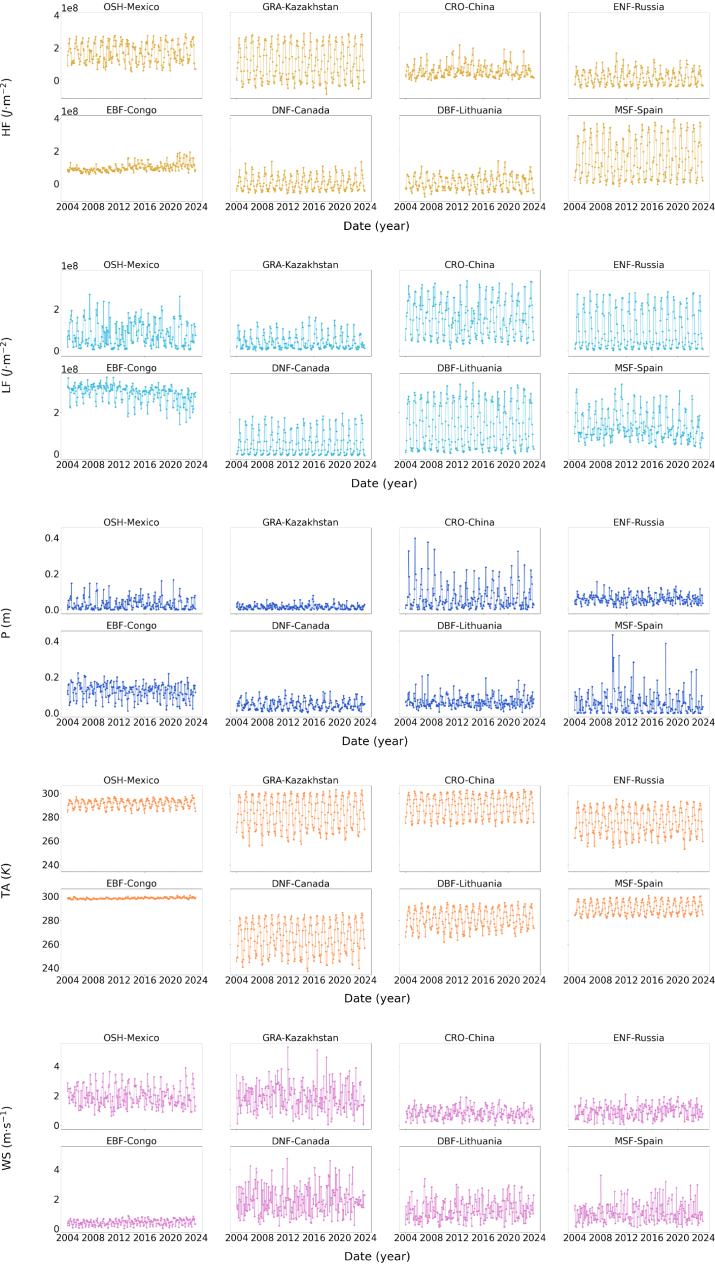
Temporal profiles of EC-GPR-TCFs predictors (2004–2023) on monthly temporal aggregation scale.

**Fig. A.7:**
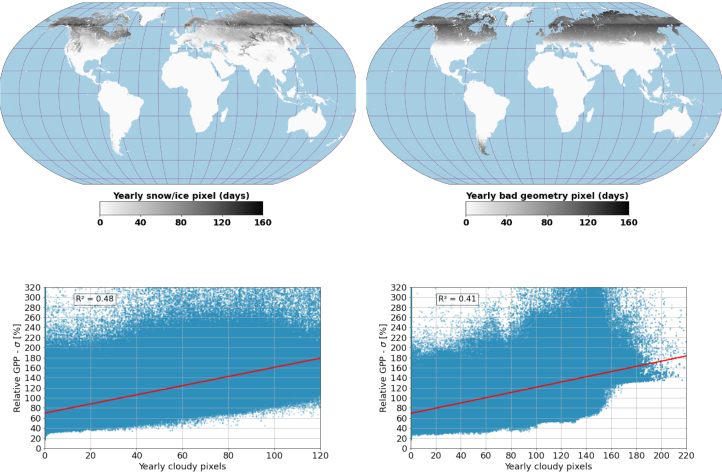
Global map distributions of factors incrementing uncertainty in the EC-GPR-TCFs models: (a) snow (yearly count of pixels with snow detection); (b) the use of alternative empirical RTM model to improve retrievals of LAI when existing extreme observation-sun geometries; (c) scatterplot of yearly count of pixels with snow detection VS. relative LAI σ (normalized by corresponding estimates); (d) scatterplot VS. number of days with bad geometries.

**Fig. A.8:**
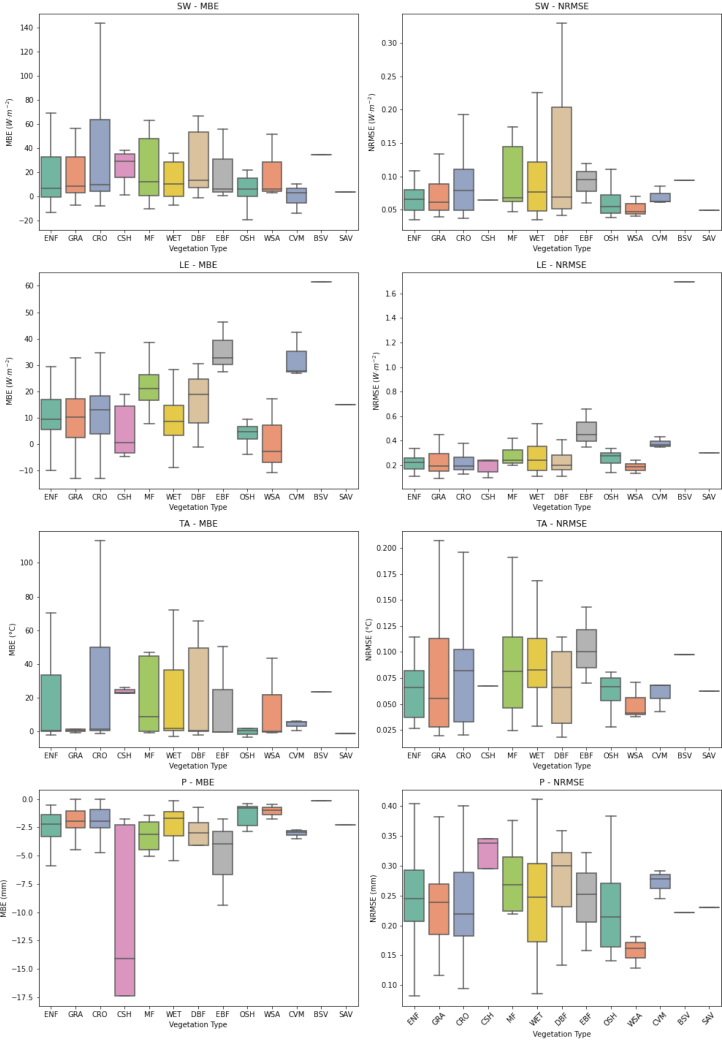
Boxplots showing nrmse (Normalized Root Mean Squared Error) and MBE (Mean Bias Error) difference metrics between ERA5-Land and EC field measurements for the time range (2019–2024). The time series for intercomparison were produced over 8-days time aggregations.

## Data Availability

The workflow/codes of this work are shared in a supplementary material section.

## Appendix A

See Fig. A.1, Fig. A.2, Fig. A.3, Fig. A.4, Fig. A.5, Fig. A.6, Fig. A.7, Fig. A.8 and Table A.1

## Appendix B Supplementary material

The codes and models developed to upscale TCFs, and open for the community, are available in the repository https://github.com/psreyes/EC-GPR-TCFs (accessed on 31 July 2024)
